# Early expression onset of tissue-specific effector genes during the specification process in sea urchin embryos

**DOI:** 10.1186/s13227-023-00210-2

**Published:** 2023-04-26

**Authors:** Shumpei Yamakawa, Atsuko Yamazaki, Yoshiaki Morino, Hiroshi Wada

**Affiliations:** 1 Institute of Zoology and Evolutionary Research, Friedrich-Shiller University Jena, Erbertstraße 1, 07747 Jena, Germany; 2 Graduate School of Life and Environmental Sciences, University of Tsukuba, 1-1-1 Tennodai, Tsukuba, Ibaraki 305-8572 Japan; 3 Faculty of Life and Environmental Sciences, University of Tsukuba, 1-1-1 Tennodai, Tsukuba, Ibaraki 305-8572 Japan

**Keywords:** Sea urchin, Differentiation, Effector genes, Gene regulatory network, Transcriptome

## Abstract

**Background:**

In the course of animal developmental processes, various tissues are differentiated through complex interactions within the gene regulatory network. As a general concept, differentiation has been considered to be the endpoint of specification processes. Previous works followed this view and provided a genetic control scheme of differentiation in sea urchin embryos: early specification genes generate distinct regulatory territories in an embryo to express a small set of differentiation driver genes; these genes eventually stimulate the expression of tissue-specific effector genes, which provide biological identity to differentiated cells, in each region. However, some tissue-specific effector genes begin to be expressed in parallel with the expression onset of early specification genes, raising questions about the simplistic regulatory scheme of tissue-specific effector gene expression and the current concept of differentiation itself.

**Results:**

Here, we examined the dynamics of effector gene expression patterns during sea urchin embryogenesis. Our transcriptome-based analysis indicated that many tissue-specific effector genes begin to be expressed and accumulated along with the advancing specification GRN in the distinct cell lineages of embryos. Moreover, we found that the expression of some of the tissue-specific effector genes commences before cell lineage segregation occurs.

**Conclusions:**

Based on this finding, we propose that the expression onset of tissue-specific effector genes is controlled more dynamically than suggested in the previously proposed simplistic regulation scheme. Thus, we suggest that differentiation should be conceptualized as a seamless process of accumulation of effector expression along with the advancing specification GRN. This pattern of effector gene expression may have interesting implications for the evolution of novel cell types.

**Supplementary Information:**

The online version contains supplementary material available at 10.1186/s13227-023-00210-2.

## Background

The multicellular bodies of animals develop from a fertilized egg through numerous cell divisions [1]. Each cell determines its cell fate and specializes during the course of the developmental process, ultimately producing various differentiated tissues composing an animal body [1, 2]. In a textbook example, specification is regarded as part of the commitment of a cell to a certain fate [1]. Specified cells have the potential to differentiate autonomously in a neutral environment such as a petri dish. Differentiation is defined as the development of specialized cells [1]. This specification, and subsequent differentiation which is generally considered to occur as an endpoint following a period of specification, have been a critical focus in the attempt to understand the genetic principles that control animal development and its evolution [1, 2].

Sea urchins are one of the best model species to investigate the regulation of differentiation as the genetic control of their cell lineages has been well investigated at single cell resolution [2, 3]. Research using sea urchins imply that capturing differentiation as an endpoint of specification may be oversimplified. For example, the pigment cells are often referred to as differentiating when they begin to express the genes for pigmentation [4, 5]. However, it is many hours later before there is evidence that they function in immunosurveillance [4–6]. Moreover, they also change their cell state and increase their motility in response to infection or wounding [6, 7]. Referring to the above examples, differentiation processes rather seem to overlap with specification and proceed without clear start and endpoint.

**Fig. 1:**
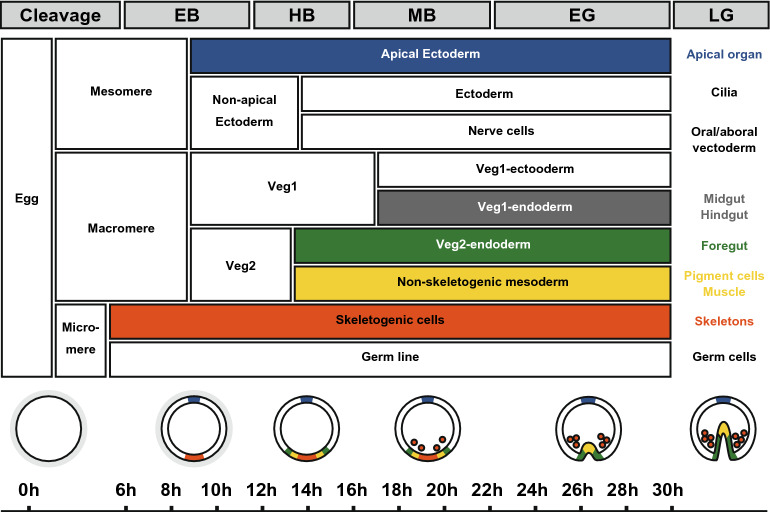
Cell fate segregation during development of the sea urchin *H. pulcherrimus*. The timing of cell fate segregation and embryonic stages were defined based on previous work using the sea urchin *S. purpuratus* [14, 25, 27, 29, 30, 36]. Embryonic stages at each developmental time (0, 6, 8, 10, ... 30 hpf) in *H. pulcherrimus* were defined with reference to the previous descriptions and our observations [22]. Based on the above information, cell fate segregation along developmental time in *H. pulcherrimus* was estimated. Egg cleavage generates three cell lineages of mesomeres, macromeres and micromeres, segregating to the apical/nonapical ectoderm, Veg1/2 and skeletogenic/germline lineages, respectively. Each lineage segregates further to establish and differentiate various tissues, such as the apical organ, cilia, mid/hindgut, pigment and skeletogenic cells. Cell lineages (top) and cellular regions in an embryo (bottom). Apical ectoderm, Veg1 endoderm, Veg2 endoderm, NSM and skeletogenic cells are highlighted in blue, gray, green, yellow and orange, respectively. *EB* early blastula, *HB* hatched blastula, *MB* mesenchyme blastula, *EG* early gastrula and *LG* late gastrula

Nevertheless, the current genetic regulatory scheme of specification and differentiation has been described according to the simplistic view of differentiation endpoint model [2, 8–10]. During the developmental process, GRN circuits of specification, which consist of complex interactions of regulatory genes such as transcription factors and signaling molecules, generate and establish distinct regulatory states in different spatial regions of an embryo [2, 11, 12]. This eventually leads to cell fate segregation in each specific cellular region (Fig. 1). In parallel with this process, a small set of regulatory genes, termed differentiation driver genes, begin to be expressed in each region [2, 11, 12]. These gene sets ultimately activate the expression of tissue-specific effector genes, which are the cohorts of genes that give rise to the biological identities of differentiated cell types [2, 11, 12]. In this model, they provided the mechanistic definition of specification and differentiation: the process to establish a cellular population with a uniform regulatory state through the interaction of regulatory genes, the installation of the expression of tissue-specific effector genes to perform cellular biological functions, respectively [2].

Previous works, however, reported that some tissue-specific effector genes are regulated directly by upstream transcription factors to promote early specification in sea urchin [2, 9, 13, 14]. For example, *Mtmmpb* (*Matrix metalloproteinase*) and *Cyp1* (*Cyclophilin1*), which function in the skeletogenesis, are directly regulated by specific transcription factors such as *Alx1* and *Ets* in skeletogenic progenitor cells, respectively [15, 16]. Thus, the *Cyp1* gene began to be expressed in the vegetal epithelium of embryos in parallel with the skeletogenic cell specification process [15]. Expression onset during the specification process was also observed in other tissues, such as endoderm cells, in which a marker gene for endoderm *Endo16* starts to be expressed at the blastula stage before gastrulation [17]. Surprisingly, some tissue-specific effector genes are reported to be expressed even before cell fate segregation. *Pks* (*Polyketide synthase*) and *Fmo* (*Flavin-containing monooxygenase*) genes are effector genes of pigment cells of nonskeletogenic mesoderm (NSM) and begin to be expressed at the blastula stage [18], well before the cell fate segregation of NSM cells. In fact, some research has already indicated that the expression of tissue-specific effector genes is also driven by early specification genes [2, 9]. The above examples do not fit well with the view of the differentiation endpoint model. In other words, the nature of differentiation regulation should be reexamined from the viewpoint of expression dynamics of tissue-specific effector genes.

Several pioneering studies have examined the expression dynamics of tissue-specific effector genes in the early development of sea urchin. Rafiq et al. 2014 compared the transcriptomes of normal embryos and embryos that lacked precursors for skeletogenic cells and comprehensively identified the tissue-specific effector genes in the skeletogenic cell lineage [19]. They found that many effector genes showed high expression levels during the late blastula to gastrula stages, when biomineralization is observed [19]. Barsi et al. 2015 obtained cell type-specific transcriptomes of six different lineages from pregastrula and early gastrula sea urchin embryos by using a combination of bacterial artificial chromosome (BAC) recombineering and fluorescence-activated cell sorting (FACS) techniques [20]. They comprehensively identified the effector genes for each cell lineage, such as skeletogenic cells, pigment cells, and apical ectodermal subdomain cells, among others [20]. However, systematic analyses of the temporal profiles of the expression of these effector genes remain to be performed.

In this study, we examined the details of the tissue-specific effector genes, specifically focusing on the expression onset of tissue-specific effector genes, in the sea urchin *Hemicentrotus pulcherrimus*. This species and the model sea urchin species *Strongylocentrotus purpuratus* diverged an estimated 9.74–14.0 million years ago [21]. Previous studies have reported the same key regulator functions and their interactions between the developmental GRNs of *H. pulcherrimus* and *S. purpuratus* [22, 23]. Based on this, we used public single-cell transcriptomic data from *S. purpuratus* to identify tissue-specific effector genes [3]. Then, we examined the expression onset of these tissue-specific effector genes through temporal transcriptomic analysis of *H. pulcherrimus* embryos (egg to early gastrula stage; 0 and 6–30 hpf at 2-h intervals). Our analysis showed the temporal overlapping expression of the tissue-specific effector genes and specification GRN components. Rather, we found that the expression of some of the effector genes commences before cell lineage segregation occurs. We propose that differentiation should be defined as an accumulation process of effector expression overlapping with specification which results in the establishment of the cellular regulatory state. Finally, our finding of dynamic expression of tissue effector genes provides interesting insight into the evolution of new cell types.

## Results

### Identification of tissue-specific effector gene cohorts in *H. pulcherrimus*

#### Extraction of non-transcription factor and non-signaling molecule genes based on domain structures

**Fig. 2:**
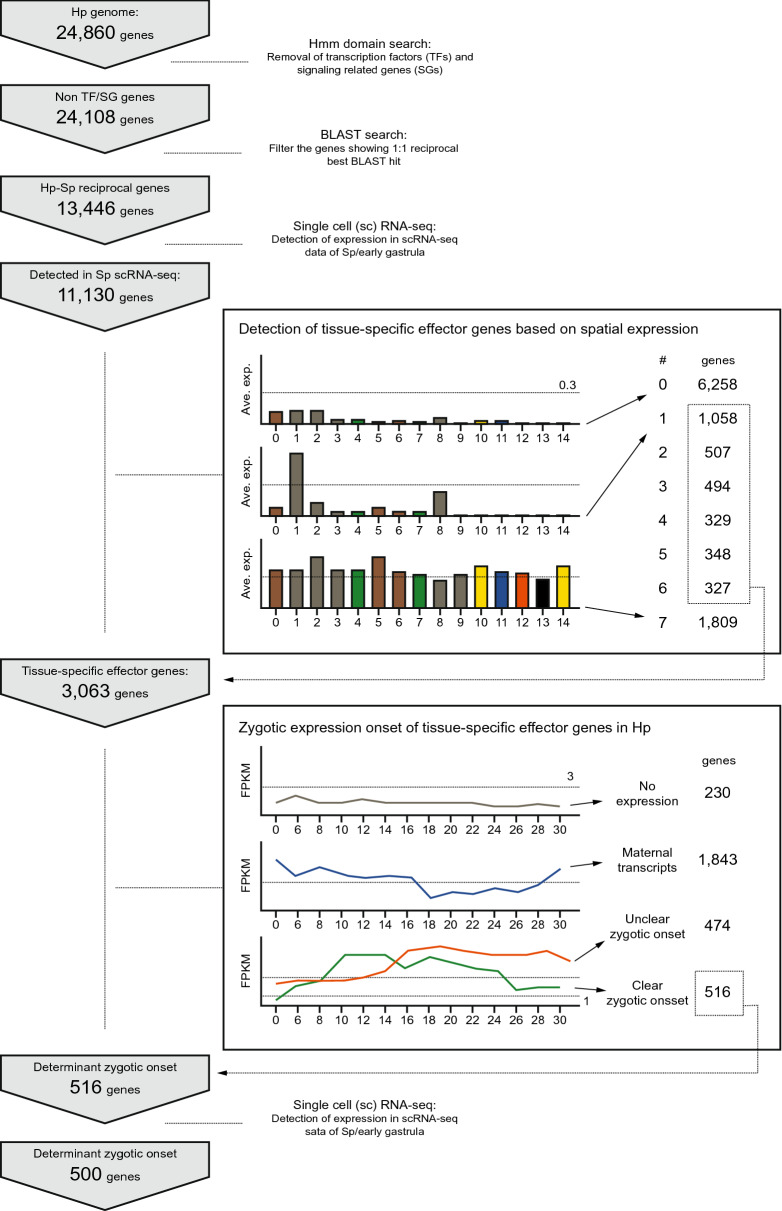
Experimental flow for screening tissue-specific effector genes. Screening of tissue-specific effector genes was conducted from the gene models of *H. pulcherrimus* genome in multiple steps. (1) Transcription factors and signaling molecules were removed based on the domain structure. (2) The genes that did not show reciprocal BLAST best hits between *H. pulcherrimus* (Hp) and *S. purpuratus* (Sp) were excluded. (3) Genes whose expression was biased to specific cell lineage(s) in single-cell RNA-seq data from *S. purpuratus* were selected. The average of expression levels (Ave. exp.) was calculated in each cell cluster (0–14) and gene. We counted the number of cell lineages in which the expression level was higher than 0.3 for each gene (#: the number of cell lineages). Three examples were shown in 0, 1 and 7 lineage(s). (4) Zygotic expression onset of the genes was determined using temporal transcriptomic data from *H. pulcherrimus*. (5) Gene annotations were checked manually to refine the sets of tissue-specific effector genes. See the text for detailed methods and results

We first attempted to systematically identify the candidate gene cohort of tissue-specific effector genes in the sea urchin *H. pulcherrimus* (experimental workflow: Fig. 2). Of 24,860 gene models that were previously identified in the genome of *H. pulcherrimus* [24], we identified 752 genes encoding transcription factors and cellular signaling proteins based on the gene domain structures. We tentatively considered the remaining 24,108 genes as the first candidate cohort of tissue-specific effector genes (Fig. 2).

This candidate list included ubiquitous housekeeping genes with roles in basic life requirements and genes that are not expressed in early developmental processes. Therefore, we next extracted the tissue-specific effector genes based on spatial expression pattern analysis.

#### Extraction of tissue-specific effector genes based on spatial expression at the early gastrula stage

Tissue-specific effector genes were expected to be expressed in specific territories of the embryo. In particular, because major specification and cell fate segregation are established at the early gastrula stage of sea urchin (Fig. 1), our target genes were assumed to be expressed in specific cell lineage(s) at this stage. Under this assumption, we examined the spatial expression patterns of the candidate genes at the early gastrula stage in a published dataset of single-cell RNA-seq of another sea urchin species, *Strongylocentrotus purpuratus* [3].

##### Dataset preparation

We first prepared and reanalyzed the single-cell RNA-seq dataset of early development of *S. purpuratus* published by Foster et al. 2020. While these authors integrated the data from various early developmental phases, we reanalyzed the data from early gastrula embryos only in order to clarify the cell states at this stage (Fig. 1). As shown in Additional file 1: Fig. S1, 15 cell clusters were reconstructed (Additional file 1: Fig. S1; apical ectoderm; Cluster 11, nonapical ectoderm; Clusters 1–3, 8 and 9, Veg1 ectoderm; Clusters 0, 5, and 6, Veg1/2 endoderm; Clusters 4, 7, nonskeletogenic mesoderm (NSM); Clusters 10, 14, skeletogenic cells; Cluster 12, germline; Cluster 13). Note that we could not technically distinguish the Veg1 and Veg2 endoderm lineage clusters from one another; thus, we identified these lineages as Veg1/2 endoderm here. See the Methods for details about the identification of cell clusters.

##### Removal of ubiquitously expressed genes to identify tissue-specific effector genes

For subsequent investigations using the dataset of different species, we first restricted our analysis to the 13,446 genes that showed reciprocal BLAST best hits between *H. pulcherrimus* and *S. purpuratus* (Fig. 2). We then investigated whether these genes had detectable expression in single-cell RNA-seq of *S. purpuratus* and found that among 13,446 genes, 11,130 had detectable expression. Finally, the average expression levels of 11,130 genes in each cluster were determined (Fig. 2).

To specify how many cell lineages at the early gastrula stage expressed each gene, the gene expression in a cell cluster was determined according to a threshold of the average expression level (Fig. 2). The median and third quantile of the average expression level of 11,130 genes in each cluster were approximately 0.0529–0.0892 and 0.232–0.356, respectively (Additional file 1: Fig. S2). Referring to these values, we set the threshold of 0.3 average expression level as the cutoff for gene expression [the validity of this threshold was tested in subsequent expression analysis (Additional file 1: Fig. S3) and gene annotation]. When multiple cell clusters were identified as belonging to the same cell lineage (ex. Clusters 0, 1, 5 and 6: Veg1 ectoderm; Clusters 2, 3, 8 and 9: nonapical ectoderm; Clusters 4 and 7: Veg1/2 endoderm; Clusters 10 and 14 for NSM lineage), the cluster that showed the highest average expression level was considered representative of that lineage (Additional file 1: Fig. S1).

We applied the above criteria to 11130 genes and found that 6258 genes did not show expression levels higher than 0.3 in any cell lineage (Fig. 2). In contrast, 1809 genes showed expression levels higher than 0.3 in all seven lineages, suggesting that these genes were housekeeping genes. We finally identified 3063 genes whose expression was restricted to specific cell lineage(s) in the early gastrula (Fig. 2). Among them, 1058 genes showed single cell lineage-specific expression, and 507 showed expression in two cell lineages. The rest of the genes showed expression in more than three lineages (Fig. 2; 494, 329, 348 and 327 genes in 3–6 lineages, respectively). We investigated the spatial expression patterns of 1058 genes whose expression was determined to be restricted to a single cell lineage according to the single-cell transcriptomic data. According to this profile, 1058 genes exhibited expression that was mainly localized to a single cell lineage (Additional file 1: Fig. S3). Note that the typical effector genes for skeletogenesis, such as *Msp130L* and *C-lectin/PMC1*, were included in this cohort. Thus, we considered that the threshold of expression level was an adequate cutoff for gene expression, and our target tissue-specific effector genes were extracted (Fig. 2).

### A number of tissue-specific effector genes begin their expression before embryonic territories are established in* H. pulcherrimus*

#### Determination of expression onset of tissue-specific effector genes during early development of H. pulcherrimus

To determine the zygotic expression onset of the 3063 tissue-specific effector genes that were screened above, we prepared whole-embryo transcriptome data from a total of 14 stages of *H. pulcherrimus*: fertilized eggs (0 hpf) and embryonic stages (6–30 hpf) from the early blastula to gastrula stage at 2-h intervals (Fig. 1). We obtained three biological replicates from different parents and calculated the average expression level (FPKM value) of each gene at each timepoint.

Expression onset was determined through the identification of the timepoint at which the FPKM value exceeded three for the first time. A total of 2833 of 3063 genes showed FPKM values higher than three at one or more of the developmental time points examined (Fig. 2; 0–30 hpf). Among these 2833 genes, 1843 genes showed FPKM values higher than three at 0 hpf, indicating that the transcripts of these genes were of maternal origin (Fig. 2). Although we aimed to identify the zygotic expression onset of these 1843 genes, we could not technically define their zygotic expression onset. The remaining 910 genes showed FPKM values higher than three for the first time between 6 and 30 hpf (Fig. 2). Of these 910 genes, 516 genes showed FPKM values of less than one at 0 hpf (Fig. 2). These genes were clearly defined as the genes that were zygotically expressed. Thus, we targeted these 516 genes in the subsequent analysis (Fig. 2).

The annotations of the 516 genes were checked manually (Additional file 1: Table S1). As representative examples, the pigmentation marker gene *Fmo* (*Fmo5_1*) was listed among the genes that were estimated to be specifically expressed in NSM. The germline marker gene *Nanos* (*Nan2*) was also screened as a germline-specific effector gene. Moreover, many skeletogenic/biomineralization marker genes, including *Clectin*s (*C-lectin*, *C-lectin/PMC1*, *Clect_13*, *Clect_25* and *Clect_76*), *Msp*s (*Msp130L* and *Msp130r1*), *Sm29* and *P16*, were included in the list of genes that were specifically expressed in skeletogenic cells. Our screening also identified a number of genes that are involved in the biological process of differentiation, such as membrane formation and cellular transportation. This validated our approach for screening tissue-specific effector genes. Notably, this gene list included 9 putative cellular signaling-related genes (*Delta*, *Dkk1*, *Eph*, *Frizz4*, *Frizz9/10*, *Hh*, *Vegf3*, *TgfbrtII* and *PlexA4_2-like*, *Tie1/2*) and 7 putative transcription factors (*Erg*, *Hnf1aL-like*, *Zmym1-like*, *Zmynd11*, *Zmynd12* and *Znhit1*). We removed these genes from subsequent analysis. Thus, the zygotic expression onset of a total of 500 genes was determined (Fig. 2).

#### Expression onset pattern of tissue-specific effector genes during early development of H. pulcherrimus

**Fig. 3:**
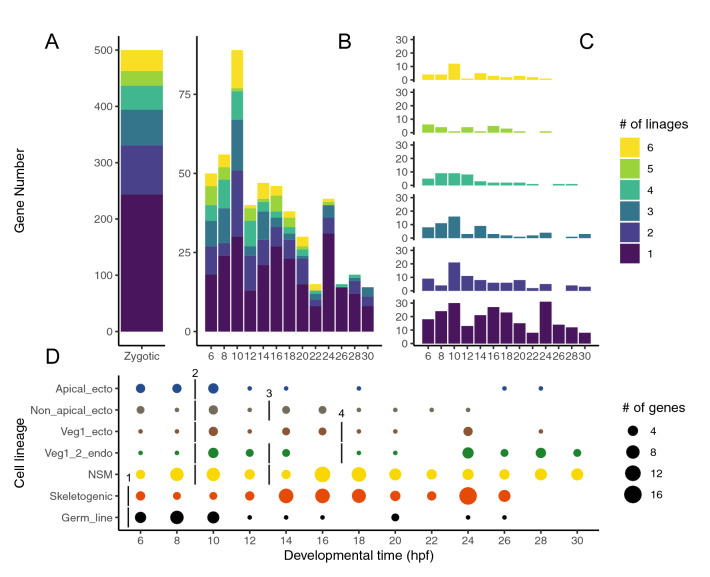
Zygotic expression onset of tissue-specific effector genes during the development of *H. pulcherrimus*. **A** Indicates the number of tissue-specific effector genes calculated as expressed in a specific number of cell lineage(s). **B** and **C** Show the number of tissue-specific effector genes with zygotic expression onset at each timepoint. The color of each bar in **A**–**C** reflects the number of cell lineages in which tissue-specific effector genes were expressed at the early gastrula stage. **B** Shows the number of genes at each developmental time stacked together, and **C** shows the number of genes expressed in each number of cell lineages separately. **D** Shows the number of tissue-specific effector genes whose expression was restricted to a single cell lineage at each zygotic expression onset time and each cell lineage (as shown in the bottom graphs in **C**). The number and cell lineages are reflected in the size and color of the circles, respectively. Bars 1–4 in **D** indicate the cell fate segregation timing with reference to Fig. 1. Bar 1: micromere to skeletogenic cells and germline; Bar 2: mesomere to apical ectoderm and macromere to Veg1/Veg2; Bar 3: nonapical ectoderm to ciliary ectoderm/nerve cells and Veg2 to Veg2 endoderm/NSM; Bar 4: Veg1 to Veg1 ectoderm/Veg1 endoderm

Of these 500 genes, 246 were expressed in a single cell lineage at the early gastrula stage (Fig. 3A). The remaining genes were found to be expressed in multiple cell lineages (Fig. 3A; 87, 63, 43, 25 and 37 genes in 2–6 cell lineages, respectively). We found that a large number of genes showed expression onset at the early stage, when cell lineage segregation occurs (6–10 hpf; Fig. 3B–C). This early expression onset was clearly observed for the genes that showed expression in multiple cell lineages (Fig. 3B–C). For tissue-specific effector genes that are expressed in a single cell lineage, the expression onset is rather broad; three main peaks of onset were detected at 10, 16 and 24 hpf (Fig. 3B–C). Nevertheless, most importantly, our observations are not consistent with the previous model of tissue-specific effector gene expression activation at the endpoint of specification GRNs after cell lineage territories are established (Fig. 3B–C).

**Fig. 4:**
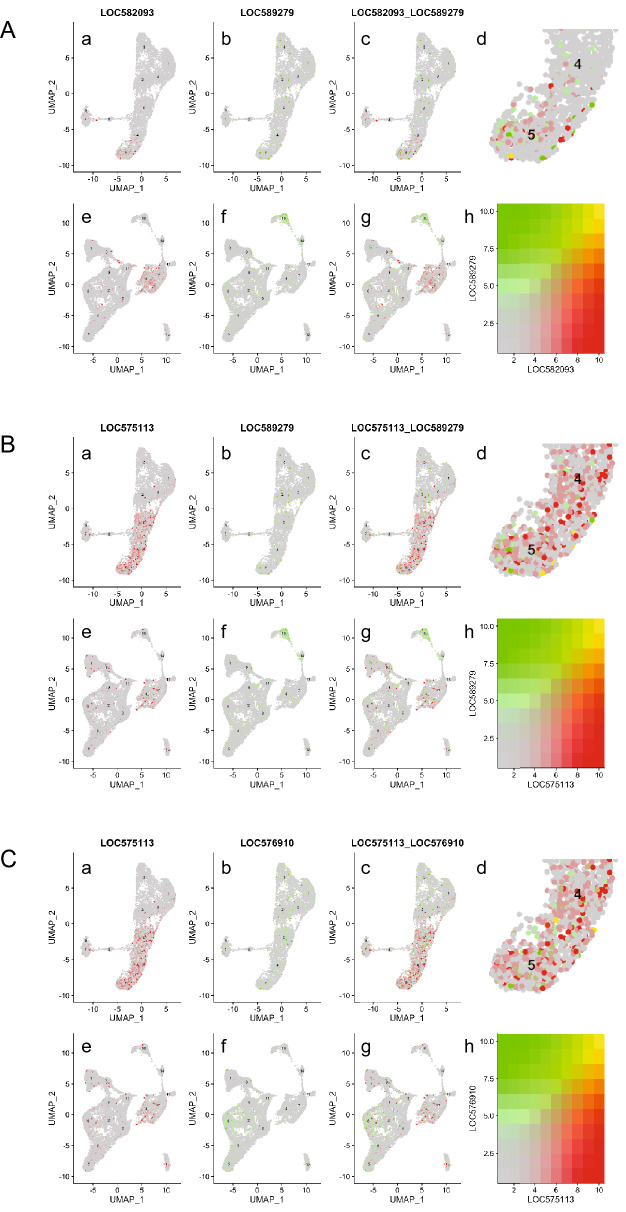
Coexpression of tissue-specific effector genes of the Veg2 endo/mesoderm and Veg1 ecto/endoderm in their precursor cells. **A**–**C** Show the expression patterns of LOC582093/*Cadherin_6* and LOC589279/*Got1*; LOC575113/*Rergl4* and LOC589279/*Got1*; and LOC575113/*Rergl4* and LOC576910/*Hypp_5866* in the UMAP projection derived from *S. purpuratus* single-cell RNA-seq data with the Seurat option FeaturePlot and its blend function. **a** and **b** Show the expression of the former and latter genes at the early blastula stage, respectively. **c** Merges the expression patterns of **a** and** c**. **d** is a magnified view of Veg2 (**A** and **B**) or Veg1 (**C**) cell clusters. **e** and **f** Show the expression of the former and latter genes at the early gastrula stage, respectively. **h** Is the color index showing the strength of the expression level and coexpression in **c** and **g**

Furthermore, we identified the spatial expression regions of the genes that showed specific expression in a single cell lineage at the early gastrula stage (Fig. 3D). For example, 19 genes began to be expressed at 6 hpf and were expressed in a single cell lineage at the early gastrula stage (Fig. 3D). We found that early onset of gene expression at 6 hpf was observed in genes of all cell lineages: apical ectoderm, 3; nonapical ectoderm, 2; Veg1 ectoderm, 1; Veg1/2 endoderm, 1; NSM, 3; skeletogenic cells, 3; and germline, 6 (Fig. 3D). Surprisingly, we identified several effector genes with expression onset before cell lineage segregation occurs. While cell lineage segregation into the Veg2 endo-mesoderm at approximately 16 hpf in the mesenchyme blastula stage (Fig. 1), some of the specific effector genes for the Veg1/2 endoderm were expressed beginning at 6 hpf (Fig. 3D). Some NSM effectors also show expression from 6 hpf (Fig. 3D). Thus, the effector genes of endoderm and NSM are also coexpressed in the precursor cells (Veg2 blastomeres). Such coexpression was also observed in Veg1 ecto-endoderm lineage. Cell lineage segregation into Veg1 ecto-endoderm occurred at approximately 14 hpf in the hatched blastula stage (Fig. 1), while some of the specific effector genes for the Veg1 ectoderm and Veg1/2 endoderm were expressed beginning at 6 hpf (Fig. 3D). Specifically, we detected the coexpression of the endodermal (LOC582093: *Cadherin_6* and LOC575113: *Rergl4*/*Ras-like estrogen-regulated, growth inhibitor like 4*) and mesodermal (LOC589279: *Got1*/*Glutamic-oxaloacetic transaminase 1*) effectors in Veg2 endo-mesodermal cells at the early blastula stage (Fig. 4). In both cases, some Veg2 cells expressed both endoderm and mesodermal effector genes whose expression was ultimately restricted to a single lineage. Coexpression of the endoderm effector LOC575113 (*Rergl4*) and ectoderm endoderm effector LOC576910 (*Hypp_5866*) was also detected in their precursor Veg1 cells (Fig. 4).

### Early expression onset of tissue-specific effector genes was validated in the analysis of representative cell lineages

Above, we extracted the expression onset pattern of the tissue-specific effector genes from the comprehensive list of sea urchin genes. We next took a complementary approach to extract tissue-specific effector genes for the representative cell lineages. We examined the marker effector genes for the NSM, skeletogenic, Veg1/2 endoderm and apical ectoderm cell lineages because the regulatory pathways in these cell types have been described in detail (Fig. 1) [2, 14, 25–28]. Then, we directly compared expression onset among early specification GRN, differentiation driver genes and tissue-specific effector genes in each lineage.

#### Extraction of marker effector genes and regulatory genes for each cell lineage

In this analysis, we extracted the tissue-specific effector gene cohort for each cell lineage using a statistical approach. We used Seurat to find marker genes of single or multiple cell cluster (s) using single-cell transcriptomic data from *S. purpuratus* (see the Methods for details). After checking the resulting gene expression patterns manually, we ultimately elucidated 53, 66, 18 and 21 tissue-specific effector genes for NSM, skeletogenic cells, Veg1/2 endoderm and apical ectoderm, respectively (Additional file 1: Table S2). We confirmed that known marker genes were included in our screening list (for example, *Msp130* and *Sm50* for skeletogenic cells and *Pks* and *Fmo* for NSM). Finally, the expression onsets were determined using the criteria mentioned above in 44, 60, 16 and 20 genes, respectively. We also extracted the transcription factors or signaling genes that compose the specification GRN and differentiation drivers in the above four lineages from the literature.

#### Expression onset of differentiation marker effector genes in four cell lineages

##### NSM

**Fig. 5:**
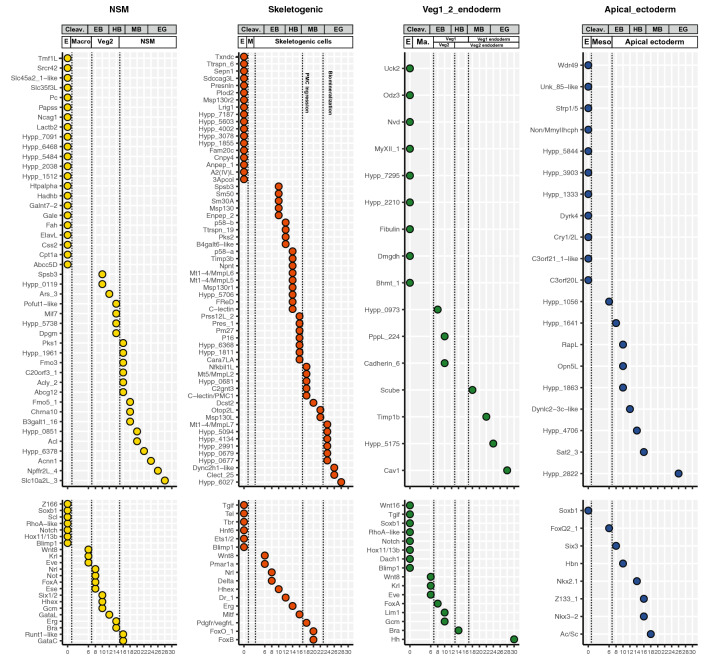
Zygotic expression onset of tissue-specific effector and specification genes in four representative cell lineages. Zygotic expression onset of tissue-specific effector genes and specification genes are shown in each representative cell lineage: **A**, **B**: NSM; C, D: skeletogenic cells; E, F: Veg1/2 endoderm; G, H: apical ectoderm (A, C, E and F: effector genes and B, D, F and H: specification genes). Each dot indicates the timing of expression onset, and dotted lines indicate the timing of cell fate segregation or morphogenesis of a developmental landmark in each cell lineage. Embryonic stages and cell fate segregation are shown in Fig. 1. *E* *egg*, *Macro/Ma* macromere, *M* micromere, *Meso* mesomere, *Cleav* cleavage

NSM cells are segregated from the Veg2 cell lineage at the hatched blastula stage (12–16 hpf; Fig. 1) and invaginate into the blastocoel from the tip of the archenteron at the late gastrula stage (Fig. 1) [26, 29, 30]. Such mesenchymal cells are called secondary mesenchymal cells (SMCs) and differentiate into various mesodermal cells, including pigment cells and coelomocytes [29, 30]. We found that early specification genes for the Veg2 lineage, such as *Eve* and *Foxa* [26, 28]*,* begin to be expressed at approximately 6–12 hpf (Fig. 5B). A previous study reported that *GataE (GataL)*, *Six1/2* and *GataC* drive the expression of differentiation effector genes [31, 32]. These differentiation driver genes were initially expressed at 12, 10 and 16 hpf (Fig. 5B). This expression onset pattern is consistent with the cell fate segregation of the Veg2 endo-mesoderm occurring at approximately 12–16 hpf.

Among the 44 tissue-specific genes that were screened for NSM, 22 marker effector genes were zygotically expressed only during 10–28 hpf. Among these, several effector genes began to be expressed in parallel with the expression of some specification genes; 11 genes were zygotically expressed at 10–14 hpf, corresponding to early and hatched blastula stages (Fig. 5A). In particular, effector genes such as *Spsb3*, *Hypp-0119* and *Ars-3* began to be expressed even before cell fate segregation at 10 or 12 hpf (Fig. 5A). The onset of the remaining zygotic genes occurred after cell fate segregation, and several genes considered to function in pigmentation were in this cohort: *Fmo3*, 16 hpf; *Psk1*, 16 hpf and *Fmo5_1*, 18 hpf (Fig. 5A). The remaining 22 effector genes were those whose transcripts were distributed in the egg (Fig. 5A). Although the zygotic expression onset of these genes was unclear in most cases, we could estimate the zygotic expression onset of several maternal genes based on the temporal expression pattern (Additional file 1: Fig. S4). Among these genes, *Abcc5D* was inferred to undergo zygotic expression onset at 10 hpf before cell fate segregation, as the expression level of *Abcc5D* decreased from 0 hpf (FPKM: 3.23) to 8 hpf (FPKM: 1.86) and then increased after 10 hpf (FPKM: 4.93; Additional file 1: Fig. S4). Therefore, our data confirmed that some tissue-specific effector genes for NSM begin to be expressed prior to the segregation of the Veg2/NSM lineage.

We further investigated the spatial expression patterns of representative effector genes (*Srcr42* and *Spsb3*) to validate that transcriptome-based analysis accurately captured the marker genes of the NSM lineage and their expression onsets. *Srcr42* transcripts were localized in eggs, and *Spsb3* was zygotically expressed in the Veg2 cell lineage before the segregation of the endo-mesoderm (Figs. 5A, B, 7A–D). Our subsequent investigation showed *Srcr42* as broadly expressed in whole eggs (Fig. 7A, B: 0 hpf) and early blastula embryos (Fig. 7A, B: 6 hpf) but specifically expressed in the NSM region after the mesenchyme blastula stage (Fig. 7A, B: 16, 24 hpf). *Spsb3* was specifically expressed in a Veg2 tier at the vegetal pole of the early blastula at 10 hpf, before the Veg2 endo-mesoderm was clearly segregated (Figs. 1, 7C, D). *Spsb3* was then expressed in the Veg2 cell lineage at 12, 18 and 24 hpf (Fig. 7). These expression patterns were clearly consistent with the results of our transcriptome-based determinations of expression onset.

##### Skeletogenic cells

The precursors of skeletogenic cells, PMCs, are specified from micromere lineages by maternal factors, such as beta-catenin polarization at the vegetal pole of embryos during the cleavage stage (Fig. 1) [10, 14, 29]. Then, PMCs ingress into the blastocoel at the mesenchyme blastula stage and finally differentiate into skeletogenic cells through cellular aggregation and mineralization at the early gastrula stage [10, 14, 29].

As the fate of skeletogenic cells is committed at the cleavage stage, we found that early specification genes such as *Wnt8*, *Pmar1a*, *Nrl* and *Delta* [10, 14] began to be expressed at 6–8 hpf, the earliest timepoint in our dataset (Fig. 5D). Genes such as *DriI, Erg*, and *FoxB* were reported to function as differentiation driver genes to activate tissue-specific effector genes in skeletogenic cell lineages [15]. The expression onset of these genes was observed at 12–20 hpf (Fig. 5D). During this timeframe, we detected that some effector genes, such as *Spsb3*, *Sm50* and *Enpep_2,* began to be expressed at 10 hpf, corresponding to the early blastula stage (Fig. 5C). The expression onset of effector genes such as *Pks2*, *Timp3b* and *Prss12L_2* occurred at 12–16 hpf, the time at which differentiation driver genes began to be expressed (Fig. 5C). Other genes, such as *Otop2L* and *Hypp-5094,* began to be expressed after establishment of the regulatory state, at 22–28 hpf (Fig. 5C). We also found that transcripts of some effector genes were maternally localized in eggs, as observed for NSM (Fig. 5C; 18 of 61 genes), and the zygotic expression onset could be estimated for only two of these genes (Additional file 1: Fig. S4; *Sdccag3L*: 24 hpf, *Anpep_1*: 16 hpf). In summary, early expression and accumulation of effector genes were also observed in skeletogenic specification process.

**Fig. 6:**
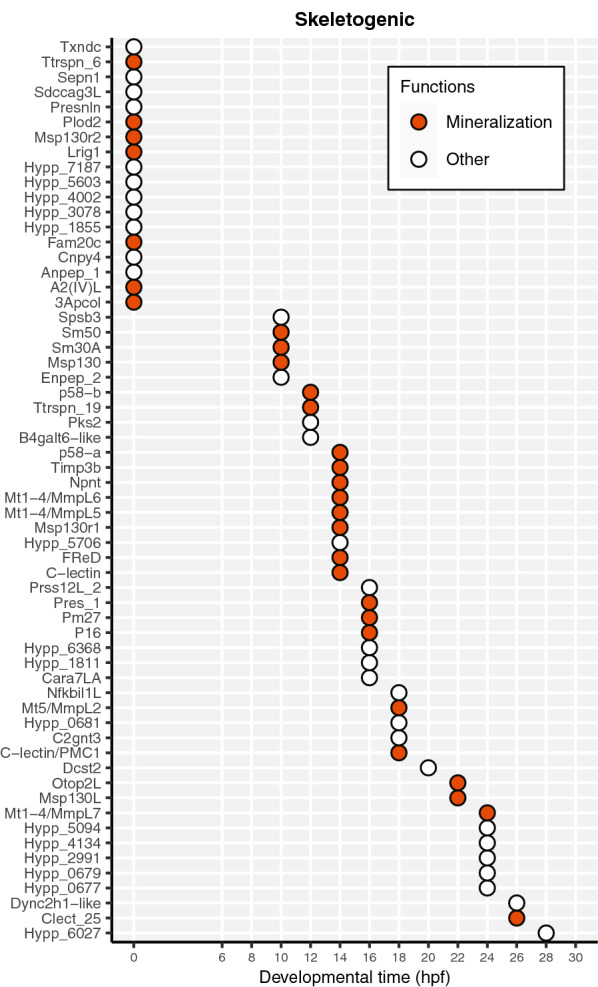
Zygotic expression onset of biomineralization genes in skeletogenic cells. Zygotic expression onset of skeletogenic effector genes that are expected to function in the mineralization process is highlighted in red. The dataset is the same as that shown in Fig. 5C. White dots represent the genes whose functions are unknown or not involved in the biomineralization process

We also clarified the expression onset of effector genes that were previously identified to function in the biomineralization process (i.e., *Msp130*, *Plod2*, *Sm50* and *3Apcol*) [33, 34]. Some biomineralization genes began to be expressed at approximately 12–16 hpf (Fig. 6), and there was no tendency for biomineralization genes to begin to be expressed only after later stages, such as the mesenchyme blastula or early gastrula stage. We also found that genes such as *Ttrspn*, *Plod2*, *Msp130r2*, *Fam20c*, *A2(IV)* and *3Apcol* were maternally distributed in eggs (Fig. 6). These findings indicated that even effector genes with specific functions in biomineralization began to be expressed prior to regulatory state establishment.

**Fig. 7:**
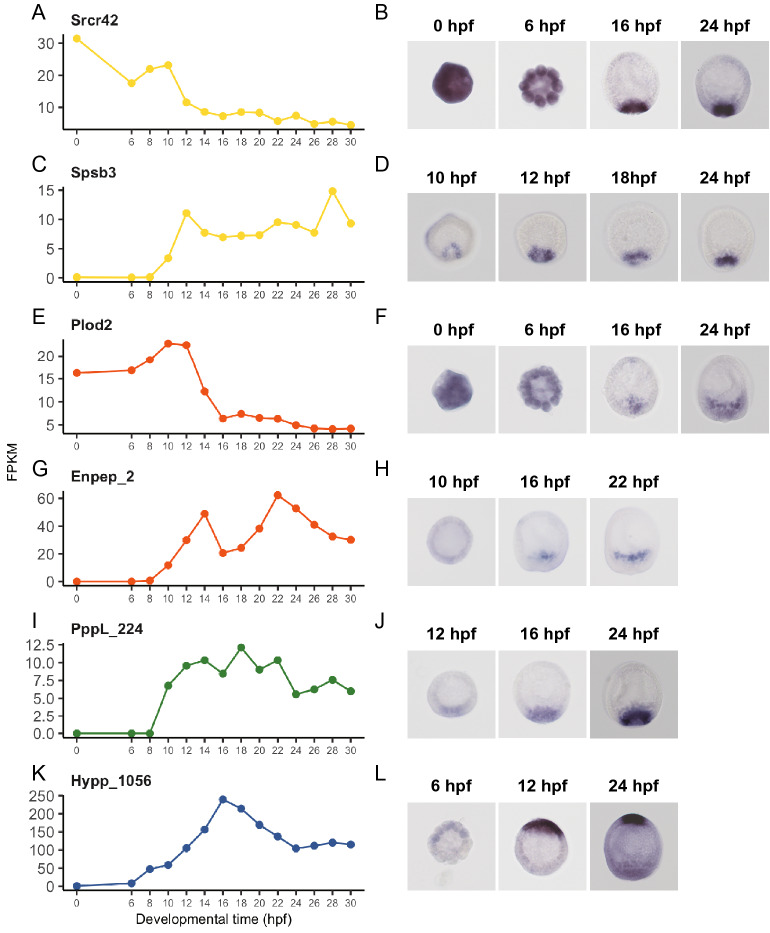
Spatial expression pattern of the representative tissue-specific effector genes. Temporal (**A**, **C**, **E**, **G**, **I**, **K**) and spatial (**B**, **D**, **F**, **H**, **J**, **L**) expression patterns of each gene were, respectively, obtained from transcriptome data and whole-mount in situ hybridization (**A**, **B**: *Srcr42*; **C**, **D**: *Spsb3*; **E**, **F**: *Plod2*; **G**, **H**: *Enpep_2*; **I**, **J**: *PppL_224*; **K**, **L**: *Hypp_1056*). Developmental timepoints for in situ hybridization are indicated above each photo of **B**, **D**, **F**, **H**, **J** and **L**

We investigated the spatial expression patterns of two representative genes for skeletogenic cells: *Plod2* and *Enpep_2*. Consistent with the FPKM values, ubiquitous expression of *Plod2* was detected in eggs and early blastula embryos (Fig. 7E, F: 0 and 6 hpf, respectively). Specific expression in skeletogenic cells was also observed in the mesenchyme blastula and early gastrula (Fig. 7F: 16 and 24 hpf, respectively), suggesting that maternal transcripts persisted until the early developmental phase and that zygotic expression specifically occurred in skeletogenic cells. We also detected the specific expression of *Empep2* in skeletogenic cells from the early blastula to early gastrula stage (Fig. 7G, H) and confirmed that the calculated expression level reflected the endogenous expression profile.

##### Veg1/2 endoderm cells

Figure 5E shows the expression onset of Veg1/2 endoderm effector genes. As mentioned above, the Veg2 cellular population segregated to NSM and Veg2 endoderm at the hatched blastula stage (Fig. 1) [26, 29, 30]. Veg1 endoderm cells develop from the Veg1 cell lineage at the mesenchyme blastula stage (Fig. 1) [26, 30]. We detected that the expression of early specification genes such as *Eve* and *Foxa* and differentiation driver genes such as *Bra* was initiated at 6–8 hpf and 14 hpf, respectively (Fig. 5F) [26, 28]. Therefore, the regulatory state of Veg1/2 endodermal cells was estimated to be established at approximately 10–14 hpf.

Our data also showed that approximately half of the effector genes for the Veg1/2 endoderm were those whose transcripts were maternally distributed in eggs (Fig. 5E, 9 of 16 genes). Their zygotic expression onset could not be calculated. On the other hand, three genes (*Hypp_0973*, *PppL_224*, and *Cadherin_6*) began to be expressed at 8–10 hpf, at which time the Veg1/2 endodermal regulatory state was not clearly segregated (Fig. 5E). Thus, consistent with the results for NSM, both endodermal and mesodermal effector genes commonly initiate expression in their precursor Veg2 cells.

We detected the coexpression of *Spsb3* (a mesodermal effector gene: Fig. 7D) and *Pppl_224* (an endoderm effector gene: Fig. 7J) in Veg2 cells at the early blastula stage, at which the Veg2 lineage was not clearly segregated (Fig. 7J). We found that the expression of *Pppl_224* could be observed in the epithelium of the vegetal pole, including the Veg2 region, at the early blastula stage (12 hpf: Fig. 7J), similar to the expression of *Spsb3* at the same stage (10 hpf: Fig. 7D). *Pppl_224* continued to be expressed until the early gastrula stage and finally localized specifically to the endodermal region at the early gastrula stage (20, 24 hpf: Fig. 7J). These spatial expression patterns suggested that the expression of tissue-specific effector genes for endoderm and mesoderm commence simultaneously in their precursor cells.

##### Apical ectodermal cells

Finally, we investigated the expression onset of tissue-specific effector genes of the apical ectoderm (Fig. 5G, H) and found expression onset patterns similar to those of other lineages. The cell fate of the apical ectoderm is clearly segregated from mesomeres in the early blastula stage (Fig. 1), although several regulators, such as the *Foxq2* gene, began to be expressed in the apical ectodermal region at approximately the 32-cell stage [27, 35, 36]. In the later stages, such as the hatched blastula stage, neural progenitor cells develop from the apical ectoderm [36].

We found that early specification genes such as *Foxq2*, *Six3* and *Hbn* begin to be expressed at 6–10 hpf [27]. Differentiation driver genes such as *Z133 (Z133_1)* and *Ac/Sc* began to be expressed at 16 and 18 hpf, respectively [27]. Our data also indicated that the onset of expression of some effector genes (*Hypp_1056*, *Hypp_1641*, *RapL*, *Opn5L* and *Hypp_1863*) occurred at 6–10 hpf, at which the cell fate segregation of mesomere occurs (Fig. 5G). Eleven genes were maternal genes whose transcripts were detected in eggs, and their zygotic gene expression onset could not be determined. We finally observed that the gene *Hypp-1056*, one of the screened effector genes, was expressed in the apical ectoderm in the early, hatched blastula and early gastrula stages (Fig. 7L: 6, 12 and 24 hpf, respectively). Therefore, even in the apical ectodermal region, our data suggested that many effector genes began to be expressed before the regulatory state and cell fate was clearly established.

Finally, we note that the expression onset of some effector and regulator genes has already been reported in sea urchin species such as *S. purpuratus*, *Lytechinus variegatus* and *Paracentrotus lividus *[18, 19, 27, 28, 31, 37–70]. We have summarized the expression onset of these genes in Additional file 1: Table S3. The results validated that most of the genes showed similar expression onset in these sea urchin species compared to *H. pulcherrimus*. Thus, it is suggested that the early expression onset pattern shown in this study is not specific to *H. pulcherrimus*, but is generally observed in sea urchin species.

## Discussion

According to the differentiation endpoint model, the GRN has been proposed as a regulatory scheme underlying early specification and differentiation in sea urchins [2, 10–12, 28]. In this scheme, transcription factors in early specification GRNs, together with some signaling molecules, generate cell lineage territories in embryos and subsequently activate the expression of differentiation driver genes, which also include additional transcription factor genes [2, 11, 12]. Finally, the transcription factors among the differentiation driver genes subsequently regulate the expression of tissue-specific effector genes in each territory [2, 11, 12].

However, contravening this model, the expression onset of some effector genes was reported to be directly regulated by early specification genes [2, 9, 13, 14]. This study suggests that the early expression of some effector genes is not exceptional but rather a general observation in various lineages of sea urchin embryos. In this study, we first comprehensively extracted the tissue-specific effector genes for seven cell lineages (Fig. 3). Although the timing of cell fate segregation is different among cell lineages, tissue-specific effector genes of any lineage commonly begin to be expressed around the blastula stage (Fig. 3). Moreover, we validated the early expression of several tissue-specific effector genes during specification process in representative cell lineages (Fig. 5). In some cell lineages, such as Veg1 and Veg2, tissue-specific effector genes begin to be expressed before cell fate segregation (Figs. 3, 4, 5, 6, 7). For example, both tissue-specific effector genes for NSM and Veg2 endoderm are expressed in their precursor Veg2 cells (Figs. 4, 7). Overall, tissue-specific effector genes generally begin to be expressed in parallel with specification GRN and even before cell fate segregation during the early development of sea urchins. This finding is not consistent with the differentiation endpoint model. We rather support that differentiation overlaps with specification processes and proceeds without clear start and endpoint as mentioned in the examples of pigment cells. In other words, the differentiation and its regulatory scheme should be conceptualized as a seamless process of accumulation of effector expression along with the advancing specification GRN.

Here, we discuss some biological impacts of this early onset of effector gene expression. The first question may be whether effector gene expression in the early phase of differentiation can be harmful. Although we do not have any evidence of when the proteins encoded by effector genes are produced, our observations suggest that the early expression of tissue-specific effector genes is modest or even negligible. The presence of biomineralization matrix proteins in cells during the early differentiation process can be tolerated. Alternatively, the effects of expression may be suppressed by an active degradation mechanism. Recently, it has been recognized that active transcript degradation mechanisms are involved in specification and differentiation [71, 72]. For example, it has been reported that the interposition of specific microRNAs controls the patterning of the larval skeleton in sea urchin embryos [73, 74]. Thus, the mRNAs of tissue-specific effector genes may be selectively degraded or translationally controlled during the early specification process. We finally want to note the possibility that some effector genes are expressed prior to cell fate segregation to perform specific roles in the biological process of asymmetric fate segregation. In the future, a comprehensive genetic control model based on the specification GRN and degradation mechanism should be constructed by focusing on the expression onset of tissue-specific effector genes. In this respect, it is worth noting that a large number of tissue-specific effector genes were deposited as maternal RNAs. Generally, in early animal development, many transcripts and proteins are deposited into oocytes [75]. These transcripts and proteins function to maintain basic cell functions until zygotic gene activation occurs [75–77]. Considering that tissue-specific effector genes have specific functions in differentiation, such as skeletogenesis and pigmentation, the maternal transcripts of effector genes are unlikely to function in any biological processes during early developmental stages such as cleavage stages. For example, some maternal transcripts may be localized to a specific cell lineage with selective degradation from all other lineages.

Another implication of the early onset of effector expression may be the evolution of new cell types or functions. We found that some effector genes are coexpressed in precursor cells before cell lineage segregation occurs. This phase of early effector gene expression may provide test cases for novel repertoires of gene expression to perform novel cell functions. It is not rare for the developmental processes of certain cell types to be altered during evolution [78]. For example, echinoderm larval skeletogenesis is likely to have arisen from the co-option of adult skeletogenesis for the larval stage in the sea urchin and brittle star lineage [79, 80]. This co-option allows a developmental process in which biomineralization cells and mobile mesenchyme cells are derived from precursor cells. Then, the precursor cells coexpress the effector genes for biomineralization and those for mobile mesenchyme cells. This may have contributed to the acquisition of the unique larval skeleton. Therefore, such cases of modification of developmental processes may provide their own combination of effector gene expression and provide occasions to explore novel cell functions. In other words, the early specification process may not only serve to provide differentiation driver genes for certain lineages; in an evolutionary sense, it can also function as a showcase of new cell functions.

## Conclusions

Differentiation processes underlying animal development have attracted critical interest to understand how animal morphology evolves. Pioneer studies on sea urchin GRN established a simplistic regulatory model which capture differentiation as the endpoint of specification process. This study reexamined the differentiation endpoint model, and found that gene cohorts of tissue-specific effector genes show expression onset patterns that are not well-consistent with the previous regulatory scheme. We suggest that differentiation processes are more dynamic than previously proposed, and imply that the dynamic GRN nature drives evolution of new cell types.

## Methods

### Preparation of transcriptome data and calculation of expression level

Adult specimens of *H. pulcherrimus* were collected around Tateyama (Chiba Prefecture, Japan). Artificial fertilization was conducted with reference to previous works [81]. Embryos from three different parents were obtained and cultured using artificial seawater (commercially purchased as Marine Art BR, Osaka Yakken Co, Osaka, Japan) at 14 °C. Total RNA was extracted from living eggs (0 hpf) and embryos (6, 8, 10, ..., 30 hpf) in each replicate using TRIzol reagent (Thermo Fisher Scientific, Massachusetts, U.S.) and then purified with the RNeasy kit (Qiagen, Hilden, Germany). Preparation of paired-end libraries and sequencing (150 bp) on a NovaSeq 6000 were performed by Novogene. The raw reads were deposited in the DDBJ Sequence Read Archives (DRA015433). The quality of raw reads was evaluated by FastQC (version 0.11.5; https://www.bioinformatics.babraham.ac.uk/projects/fastqc/) and further filtered by Trimmomatic (version 0.38) [82]. The gene models of *H. pulcherrimus*, which were previously published by Kinjo et al., 2018, were used as a reference for the calculation of expression levels over developmental time [24]. Read mapping and FPKM calculation were conducted using Rsem/bowtie2 (version 1.2.28) [83]. The FPKM values of each gene in three biological replicates at each developmental timepoint were averaged for the subsequent analysis. This dataset is available in the supplementary file (see Availability of data and materials).

### General description of bioinformatic analysis and data visualization

We mainly used the R language (version 4.1.3) and its packages, such as Tidyverse (version 1.3.2), for the analysis of transcriptome data [84, 85]. Data processing was also conducted using the Unix standard command line, for example, AWK in bash/zsh. The ggplot2 (in Tidyverse) and gt packages were utilized to generate graphs (Figs. 3, 5, 6, 7 Additional file 1: S2, S4) and tables (Additional file 1: Tables S1, S2), respectively [85, 86]. Original figures were processed using Adobe Illustration without modifying the results. Code (R scripts), datasets, and original figures are provided in the supplemental files.

### Analysis of single-cell RNA-seq data of *S. purpuratus*

#### Dataset preparation and cell clustering

We obtained the raw data of single-cell RNA-seq of *S. purpuratus* early blastula (EB) and early gastrula (EG) embryos from NCBI GEO (GSE149221; GSM4494541: SpEB/early blastula stage; GSM4494544: SpEG/early gastrula) [3]. All data were further processed using Seurat (version 3.2.1) [87]. Filtering, normalization, feature selection, dimension reduction and cell clustering were performed with reference to the online manual, which was provided by the Seurat developer (https://satijalab.org/seurat/archive/v3.2/pbmc3k_tutorial.html) [87]. Essentially, we used the default values to analyze these data in the same way as the authors who published the raw *S. purpuratus* single-cell RNA-seq data [3]. A total of 10 and 15 cell clusters were found in the EB and EG, respectively (Additional file 1: Fig. S1A, B). Each cluster was annotated according to the expression patterns of the following marker genes with previously characterized expression: Apical ectoderm [35, 88]: *Foxq2* (foxq2), *Nkx2.1* (NK2.1); nonapical ectoderm [25]: *Six3* (LOC576281), Emx (LOC577702), *Unvn* (LOC373488), *Lim1* (Lim1), *Gsc* (Gsc) and *FoxG* (FoxG); Veg1 ectoderm [25]: *Eve* (eve), *Vegf3* (LOC100889860); Veg1 endoderm [25]: *Eve* (eve), *Hox7* (Hbox7); skeletogenic cells [14]: *Alx1* (Alx1), *Sm50* (SM50); NSM [28]: *Gcm* (gcm), *GataE* (GATAe); Veg2 endoderm [28]: *Blimp1/Krox* (blimp1/krox), *FoxA* (FoxA); germline [89]: *Nanos* (Nanos2). The expression of these genes was plotted on the uniform manifold approximation and projection (UMAP) projection of EB and EG data (Additional file 1: Fig. S1C).

#### Spatial expression analysis for screening of tissue-specific effector genes

The spatial expression of the candidate tissue-specific effector genes was investigated using the EG data. We used the dotplot option in Seurat to obtain the comprehensive expression data (average expression in each cluster) of each gene [87]. The expression matrix was further processed to show the distribution of expression levels in each cluster (Fig. S2) and the localization of expression of each gene (Additional file 1: Fig. S3). Coexpression of Veg2 endo-mesoderm and Veg1 ecto-endoderm effector genes was investigated using the option FeaturePlot and its blend function with the default threshold (Fig. 4) [87].

#### Extraction of marker genes of representative clusters

To obtain the list of marker genes for apical ectoderm, Veg1/2 endoderm, NSM and skeletogenic cells, the FindMarkers option in Seurat was used [87]. Using this option with default parameters, we first extracted the marker genes of cluster(s) 12, 4/7, 10 and 11. From this list, the top 100 genes with the highest p values were extracted. Then, these genes were further filtered by domain search for transcription factors/signaling machinery and BLAST reciprocal hit between *S. purpuratus* and *H. pulcherrimus* as described below. Finally, we manually reviewed and selected their spatial expression patterns (Additional file 1: Table S2).

### Determination of zygotic expression onset of tissue-specific effector and transcription factor genes in *H. pulcherrimus*

Tissue-specific effector genes were extracted from two experimental flows as described in the text (screening from comprehensive genomic dataset and extraction of the marker genes of representative cell lineages). Transcription factors were extracted with the domain search described below. Expression onset was investigated as the earliest time at which a FPKM value threshold was met during the developmental time (0, 6–30 hpf). Specifically, for each target gene, we specified the time at which the FPKM value exceeded three for the first time as the time of expression onset. We could not technically distinguish maternal and zygotic transcripts in this study. Thus, zygotic expression onset was determined only for those genes whose FPKM values were less than one at 0 hpf. In exceptional cases, we defined the zygotic expression onset of the genes that did not meet this criterion via manual analysis of representative cell lineages (Additional file 1: Fig. S4). Specifically, we manually defined the zygotic expression onsets in cases with (1) a decrease in FPKM values in early stages, such as 6–10 hpf, and (2) an increase in FPKM values just after these stages.

### Other bioinformatic analyses

#### Domain search

Domain searches of transcription factors and signaling-related genes were carried out using HMMER search (version 3.3.2) [90]. Specifically, the following hmm domains were prepared to extract transcription factors: ARID (PF01388.17), AT_hook (PF02178.15), Basic (PF01586.12), CUT (PF02376.11), DM (PF00751.14), Ets (PF00178.18), Forkhead (PF00250.14), GATA (PF00320.23), GCM (PF03615.11), HLH (PF00010.22), HMG_box (PF00505.15), Hairy_orange (PF07527.9), Homeobox (PF00046.25), Hormone_recep (PF00104.26), OAR (PF03826.13), P53 (PF00870.14), P53_tetramer (PF07710.7), PAX (PF00292.14), Pou (PF00157.13), HPD (PF05044.8), RHD_DNA_bind (PF00554.18), Runt (PF00853.15), SCAN (PF02023.13), SIM_C (PF06621.8), SRF-TF (PF00319.14), T-box (PF00907.18), TBX (PF12598.4), TF_AP-2 (PF03299.10), TF_Otx (PF03529.9), bZIP_1 (PF00170.17), bZIP_2 (PF07716.11), zf-C2H2 (PF00096.22), zf-C2HC (PF01530.14) and zf-C4 (PF00105.14). The following hmm domains were for signaling-related genes: AMH_N (PF04709.8), CSF-1 (PF05337.7), Cbl_N (PF02262.12), Cbl_N2 (PF02761.10), Cbl_N3 (PF02762.10), CheW (PF01584.15), DIX (PF00778.13), Dishevelled (PF02377.11), FGF (PF00167.14), Focal_AT (PF03623.9), G-gamma (PF00631.18), GM_CSF (PF01109.13), Hpt (PF01627.19), IL11 (PF07400.7), IL12 (PF03039.10), IL2 (PF00715.13), IL3 (PF02059.11), IL4 (PF00727.14), IL5 (PF02025.11), IL7 (PF01415.12), MCPsignal (PF00015.17), NPH3 (PF03000.10), Olfactory_mark (PF06554.8), PDGF (PF00341.13), PDGF_N (PF04692.9), PSK (PF06404.8), PTN_MK_C (PF01091.14), PTN_MK_N (PF05196.9), Phe_ZIP (PF08916.7), RGS (PF00615.15), Rabaptin (PF03528.11), STAT_alpha (PF01017.16), STAT_bind (PF02864.11), STAT_int (PF02865.13), TGF_beta (PF00019.16), TGFb_propeptide (PF00688.14), TRADD_N (PF09034.6) and wnt (PF00110.15). We searched for such domains in all gene models of *H. pulcherrimus* [24].

#### Reciprocal BLAST

BLAST software (version. 2.12.0 +) was downloaded from NCBI [91]. Amino acid sequences of *H. pulcherrimus* and *S. purpuratus* were obtained from HpBase and EchinoBase, respectively (Hp: HpulGenome_v1_prot.fa, Sp: GCF_000002235.4_Spur_4.2_protein.faa) [24, 92]. First, the amino acid sequence of *H. pulcherrimus* was used as the query of the blastp program against the *S. purpuratus* database. We then used each top hit gene model as the query for a subsequent blastp search using the *H. pulcherrimus* database. Among these BLAST searches, the top hit was the same as the original query for 14,080 genes. Reciprocal BLAST top hit genes are shown in the supplemental files.

### Whole-mount in situ hybridization (WMISH)

Eggs and embryos of *H. pulcherrimus* were obtained from the same cohorts used for RNA-seq as described above (0, 6, 8, 10, ..., 30 hpf) and fixed with 4% paraformaldehyde in artificial seawater. We obtained three biological replicates, and the embryos of replicate 1 are presented as the representative samples. Target gene sequences were obtained from the public database, and the genes were amplified using the following primers: Pppl_224_F, ctccgaagctgccatcgaagatattacatt; Pppl_224_R + T3, attaaccctcactaaagggaagctactctcggtgcataat; Hypp_1056_F, attcccgttatcgcttgaagatgattcgta; Hypp_1056_R + T3, attaaccctcactaaagggacaccactcgagagaattttg; Empep_F, ctcggatggagagatgacggttcccatctt; Empep_R + T3, attaaccctcactaaagggaaatgttctttggttctgcat; Srcr42_F, cgttacgtgcaatggtaatattcgcctaca; Srcr42_R + T3, attaaccctcactaaagggacttcttttccggtgcagttc; Plod2_F, catgagatcgaaaatgcagaaatggaagaa; Plod2_R + T3, attaaccctcactaaagggactacgttaatggtgtacgtg; Spsb3_F, aaaagctcgtctgcatgccgattccttcat; HpSpsb3_R + T3, attaaccctcactaaagggattgaataagcgtgtcctctt. WMISH was carried out as described in previous works [80, 93].

## Supplementary Information

Additional file 1: Fig S1. Identification of cell clusters in the single-cell transcriptomic data. Fig S2. Distribution of averaged expression levels of the candidate cohort of tissue-specific effector genes in each cell cluster. Fig S3. Spatial expression pattern of the tissue-specific effector genes whose expression was estimated to be restricted to a single cell cluster. Fig S4. Temporal expression pattern of the tissue-specific effector genes whose expression was observed at 0 hpf in the representative cell lineages. Table S1. List of screened tissue-specific effector genes. Table S2. List of marker tissue-specific effector genes.

## Data Availability

The raw reads were deposited in the DDBJ Sequence Read Archives (DRA015433). The supplementary files are provided in Figshare (10.6084/m9.figshare.21776603)
