# Supplementary material for: Early expression onset of tissue-specific effector genes during the specification process in sea urchin embryos
Source: EvoDevo. 2023 Apr 26;14:7. doi: 10.1186/s13227-023-00210-2 (PMC10131483; doi:10.1186/s13227-023-00210-2)
Supplement: Supplementary file 1 — Additional file 1: Fig S1. Identification of cell clusters in the single-cell transcriptomic data. Fig S2. Distribution of averaged expression levels of the candidate cohort of tissue-specific effector genes in each cell cluster. Fig S3. Spatial expression pattern of the tissue-specific effector genes whose expression was estimated to be restricted to a single cell cluster. Fig S4. Temporal expression pattern of the tissue-specific effector genes whose expression was observed at 0 hpf in the representative cell lineages. Table S1. List of screened tissue-specific effector genes. Table S2. List of marker tissue-specific effector genes. [file 13227_2023_210_MOESM1_ESM.zip › 13227_2023_210_MOESM1_ESM/supplement/Supplementary_figures.pdf.pdf]

Supplementary figures

Fig. S1 Identification of cell clusters in the single-cell transcriptomic data

The left panels of A and B indicate the UMAP projection of cell clusters derived from single-cell RNA-seq data of early blastula (EB) and early gastrula (EG), respectively. The numbers and colors of the UMAP projections reflect the cluster identity, and the annotation of each cell cluster is indicated in the tables at right. C shows the spatial expression patterns of the marker genes of specific cell lineages at EB (top) and EG (bottom). Each figure was generated by the FeaturePlot option in Seurat. Apical ectoderm: *Foxq2* (foxq2), *Nkx2.1* (NK2.1); nonapical ectoderm: *Six3* (LOC576281), *Emx* (LOC577702), *Unvn* (LOC373488), *Lim1* (Lim1), *Gsc* (Gsc) and *FoxG* (FoxG); Veg1 ectoderm: *Eve* (eve), *Vegf3* (LOC100889860); Veg1 endoderm: *Eve* (eve), *Hox7* (Hbox7), skeletogenic cells: *Alx1* (Alx1), *Sm50* (SM50); NSM: *Gcm* (gcm), *Six1* (Six1); Veg2 endoderm: *Blimp1/Krox* (blimp1/krox), *FoxA* (FoxA); germline: *Nanos* (Nanos2).

Fig. S2 Distribution of averaged expression levels of the candidate cohort of tissue-specific effector genes in each cell cluster

The average expression levels of tissue-specific effector genes in the candidate cohort were calculated by the analysis of single-cell RNA-seq data from the early gastrula stage. The violin plot and boxplot show the distribution of such expression levels in each cell cluster. Cell clusters are distinguished with colors. The Y-axis shows the logarithmic scale of the averaged expression level. The dotted line shows the 0.3 expression level, which was used as expression level threshold.

Fig. S3 Spatial expression pattern of the tissue-specific effector genes whose expression was estimated to be restricted to a single cell cluster

A total of 1,058 tissue-specific effector genes were estimated to be expressed in a single cell lineage at the early gastrula stage. A–I indicate the spatial expression of these genes in each cell cluster where the expression was expected (A: apical ectoderm, B: nonapical ectoderm, C: Veg1 ectoderm, D: Veg1/2 endoderm, E-F: NSM, G-H: skeletogenic cells and I: germline). A–I were obtained by dotplot in Seurat. The size and color gradation of each dot represent the number of expressed cells in each cluster and the strength of the scaled average expression level, respectively. The yellow line indicates the cell cluster(s) in which expression was expected for each cell lineage.

Fig. S4 Temporal expression pattern of the tissue-specific effector genes whose expression was observed at 0 hpf in the representative cell lineages

The temporal expression pattern of each target gene is shown. The color of the line graphs indicates the cell lineage (yellow: NSM, orange: skeletogenic, green: Veg1/Veg2 endoderm and blue: apical ectoderm). The X- and Y-axes indicate the developmental timepoint and expression level (FPKM), respectively, which were calculated from the transcriptome data. The horizontal solid line shows the FPKM value of 3, which was used as the gene expression threshold in this study.

Table S1: List of screened tissue-specific effector genes

Table S2: List of marker tissue-specific effector genes

Fig. S1

A

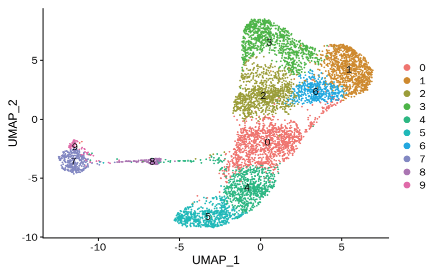

0	Veg1 / Veg2	8	Germ line
1	Non-apical ectoderm	9	Skeletogenic cells
2	Non-apical ectoderm		
3	Apical ectoderm		
4	Veg1 / Veg2		
5	Veg2		
6	Non-apical ectoderm		
7	Skeletogenic cells		

B

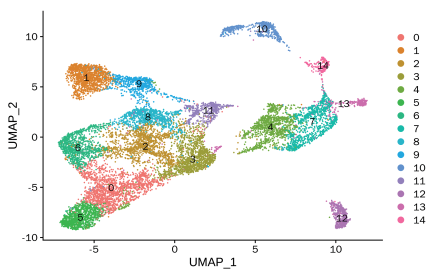

0	Veg1 ectoderm	8	Non-apical ectoderm
1	Non-apical ectoderm	9	Non-apical ectoderm
2	Non-apical ectoderm	10	Non-skeletogenic meso
3	Non-apical ectoderm	11	Apical ectoderm
4	Veg1/2 endoderm	12	Skeletogenic cells
5	Veg1 ectoderm	13	Germ line
6	Veg1 ectoderm	14	Non-skeletogenic meso
7	Veg1/2 endoderm		

C

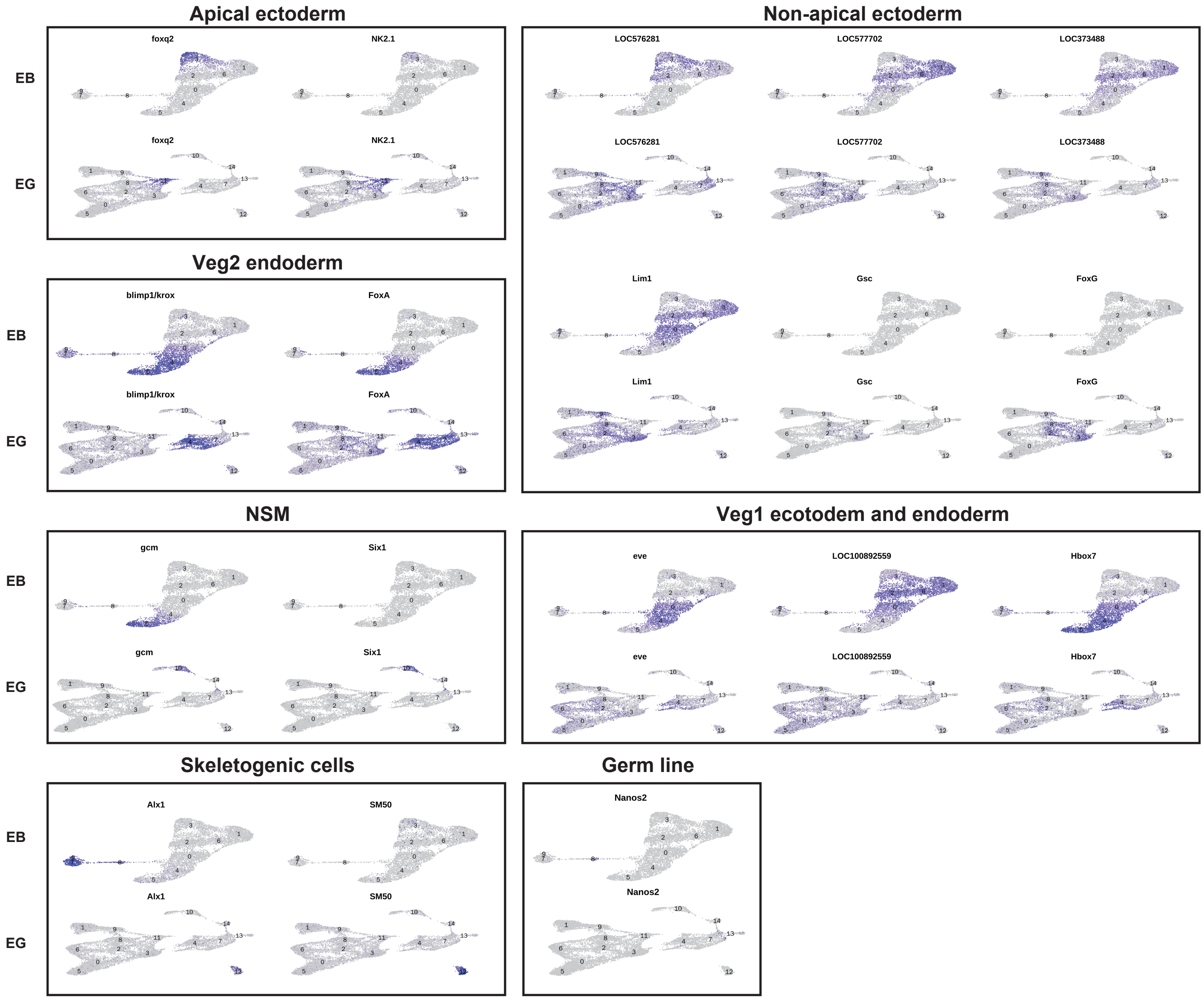

Fig. S2

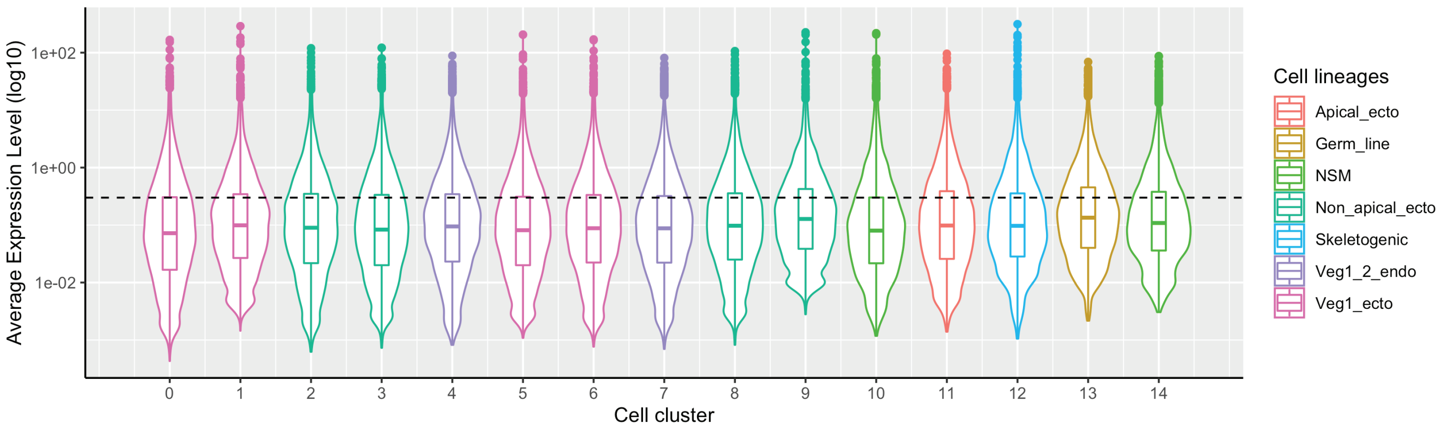
Average Expression Level (log10)
1e+02
1e+00
1e-02
0
1
2
3
4
5
6
7
8
9
10
11
12
13
14
Cell cluster
Cell lineages
Apical_ecto
Germ_line
NSM
Non_apical_ecto
Skeletogenic
Veg1_2_endo
Veg1_ecto

Fig. S3

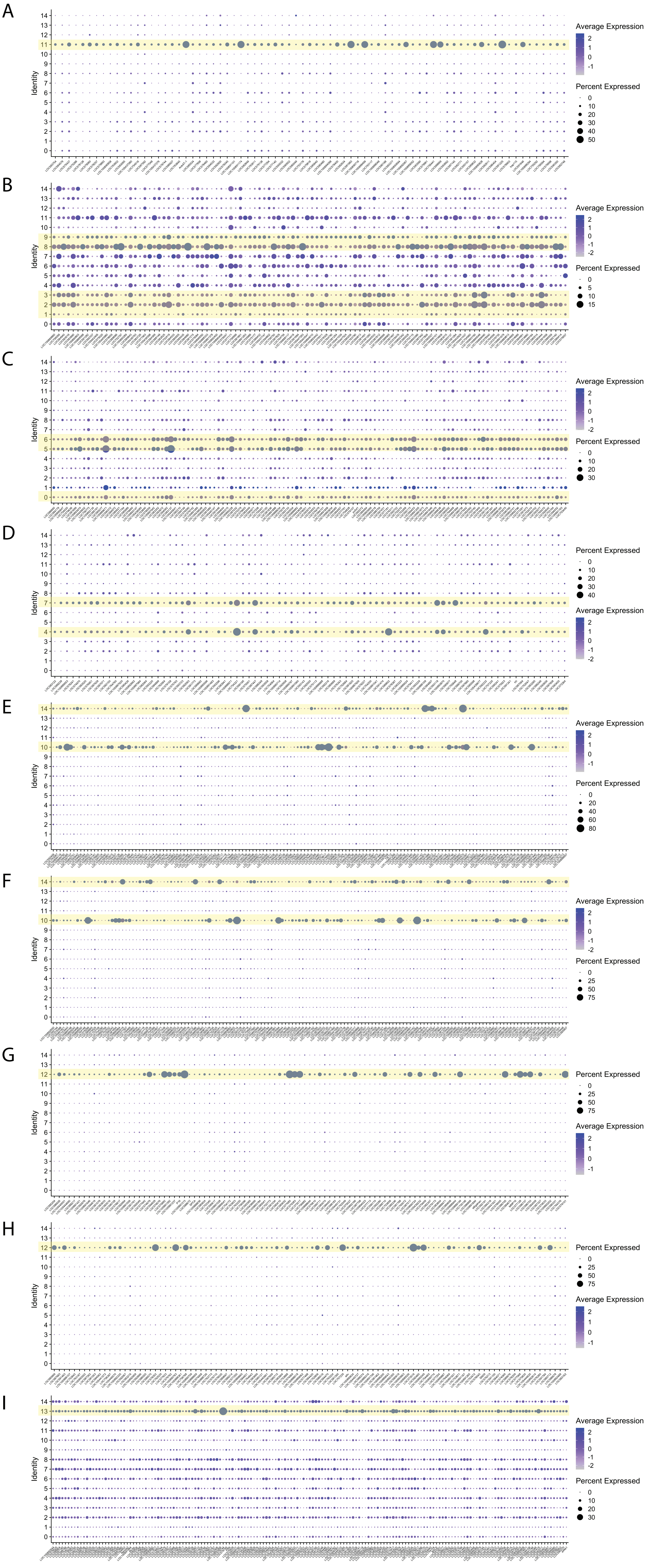
A
B
C
D
E
F
G
H
I
Identity
Average Expression
Percent Expressed

Fig. S4

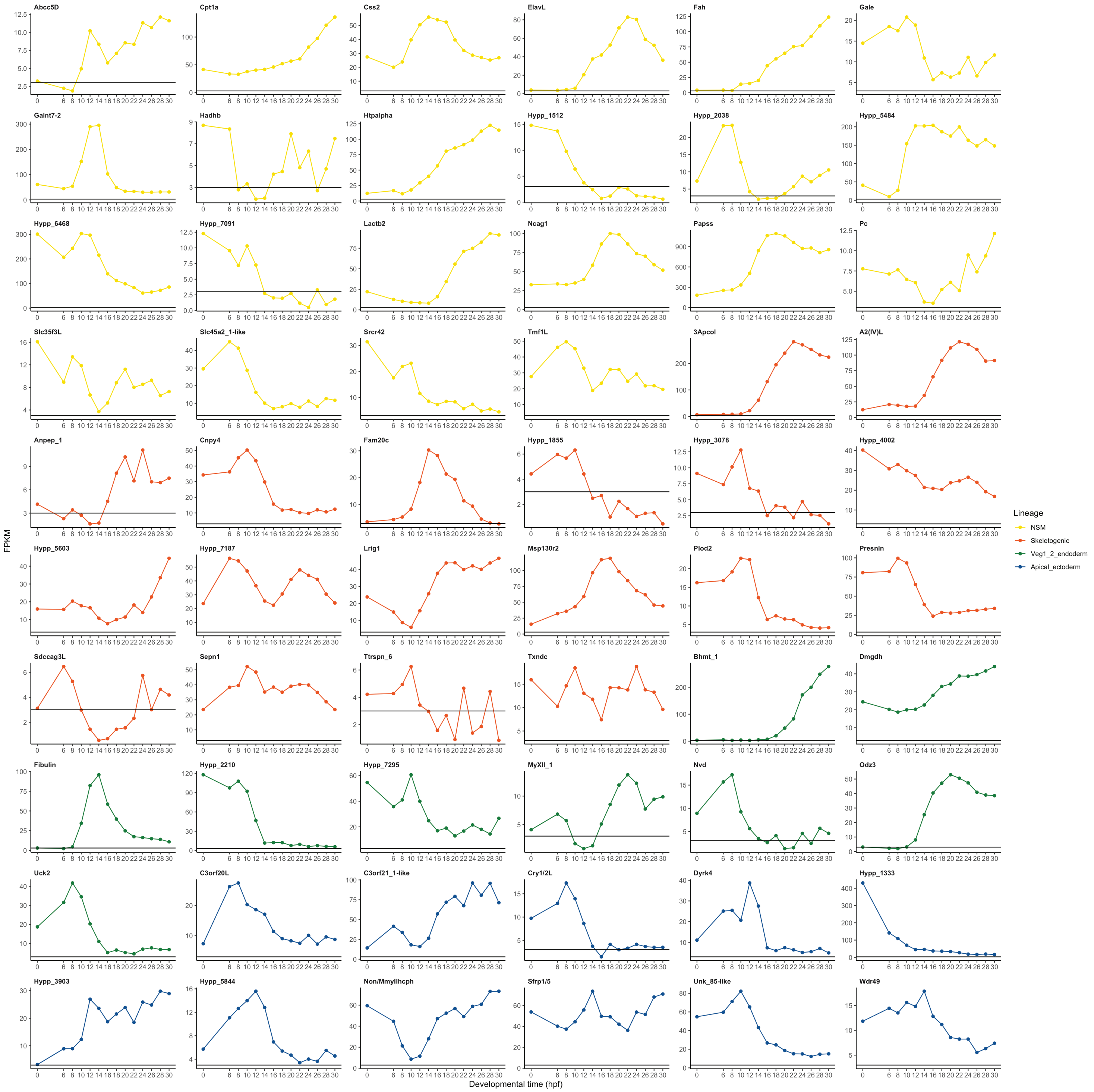
Abcc5D
Cpt1a
Css2
ElavL
Fah
Gale
Galnt7-2
Hadhb
Htpalpha
Hypp_1512
Hypp_2038
Hypp_5484
Hypp_6468
Hypp_7091
Lactb2
Ncag1
Papss
Pc
Slc35f3L
Slc45a2_1-like
Srcr42
Tmf1L
3Apcol
A2(IV)L
Anpep_1
Cnpy4
Fam20c
Hypp_1855
Hypp_3078
Hypp_4002
Hypp_5603
Hypp_7187
Lrig1
Msp130r2
Plod2
Presnln
Sdccag3L
Sepn1
Ttrspn_6
Txndc
Bhmt_1
Dmgdh
Fibulin
Hypp_2210
Hypp_7295
MyXII_1
Nvd
Odz3
Uck2
C3orf20L
C3orf21_1-like
Cry1/2L
Dyrk4
Hypp_1333
Hypp_3903
Hypp_5844
Non/Mmyllhcph
Sfrp1/5
Unk_85-like
Wdr49
FPKM
Developmental time (hpf)
Lineage
NSM
Skeletogenic
Veg1_2_endoderm
Apical_ectoderm
