# Supplementary material for: Early expression onset of tissue-specific effector genes during the specification process in sea urchin embryos
Source: EvoDevo. 2023 Apr 26;14:7. doi: 10.1186/s13227-023-00210-2 (PMC10131483; doi:10.1186/s13227-023-00210-2)
Supplement: Supplementary file 1 — Additional file 1: Fig S1. Identification of cell clusters in the single-cell transcriptomic data. Fig S2. Distribution of averaged expression levels of the candidate cohort of tissue-specific effector genes in each cell cluster. Fig S3. Spatial expression pattern of the tissue-specific effector genes whose expression was estimated to be restricted to a single cell cluster. Fig S4. Temporal expression pattern of the tissue-specific effector genes whose expression was observed at 0 hpf in the representative cell lineages. Table S1. List of screened tissue-specific effector genes. Table S2. List of marker tissue-specific effector genes. [file 13227_2023_210_MOESM1_ESM.zip › 13227_2023_210_MOESM1_ESM/supplement/Table S2.pdf]

Table S2: Gene list of marker tissue-specific effector genes

	Gene_id	Uniprot annotation
Skeletogenic		
3Apcol	HPU_12876	none
A2(IV)L	HPU_12874	none
Anpep_1	HPU_13668	Aminopeptidase N (rAPN) (Alanyl aminopeptidase) (Microsomal aminopeptidase) (CD13) (Aminopeptidase M) (APM) (Kidney Zn peptidase) (KZP)
Astacin1	HPU_06033	none
Atp8a1_1	HPU_22090	ATPase, aminophospholipid transporter (APLT), class I, type 8A, member 1
B4galt6-like	HPU_07694	Beta-1,4-galactosyltransferase 6 (Beta-1,4-GalTase 6) (Beta4Gal-T6) (b4Gal-T6) (UDP-galactose:beta-N-acetylglucosamine beta-1,4-galactosyltransferase 6) (UDP-Gal:beta-GlcNAc beta-1,4-galactosyltransferase 6) [Includes: Lactosylceramide synthase (LacCer synthase) (UDP-Gal:glucosylceramide beta-1,4-galactosyltransferase)]
C-lectin	HPU_21887	none
C-lectin/PMC1	HPU_05420	none
C2gnt3	HPU_11202	core 2 beta-1,6-N-acetylglucosaminyltransferase 3
Cara7LA	HPU_10850	none
Clect_25	HPU_06862	none
Cnpy4	HPU_05237	canopy 4 homolog (zebrafish)
Cp	HPU_10395	ceruloplasmin (ferroxidase)
Dcst2	HPU_10750	DC-STAMP domain containing 2
Dync2h1-like	HPU_16545	dynein, cytoplasmic 2, heavy chain 1
Enpep_2	HPU_10871	Glutamyl aminopeptidase (EAP) (Aminopeptidase A) (APA) (Differentiation antigen gp160)
FReD	HPU_23753	FReD/fmo5-like
Fam20c	HPU_04878	family with sequence similarity 20, member C; dentin matrix protein 4-like
Hemic1	HPU_02002	none
Hypp_0677	HPU_02487	none
Hypp_0679	HPU_02489	hypothetical protein-2386
Hypp_0681	HPU_02493	hypothetical protein-2999
Hypp_1811	HPU_06530	none
Hypp_1855	HPU_06725	hypothetical protein-112
Hypp_2991	HPU_10550	hypp-120
Hypp_3078	HPU_10844	none
Hypp_4002	HPU_13988	hypothetical protein-857; transcription factor jumonji (jmjC) domain-containing protein
Hypp_4134	HPU_14470	hypothetical protein-3018
Hypp_5094	HPU_17672	none
Hypp_5603	HPU_19357	none
Hypp_5637	HPU_19461	none
Hypp_5706	HPU_19716	none
Hypp_6027	HPU_20753	none
Hypp_6368	HPU_21914	hypothetical protein-1512
Hypp_7187	HPU_24455	hypothetical protein-3119
Lrig1	HPU_05212	leucine-rich repeats and immunoglobulin-like domains 1
Msp130	HPU_05528	none
Msp130L	HPU_05527	mesenchyme-specific cell surface glycoprotein-like
Msp130r1	HPU_05532	none
Msp130r2	HPU_17194	none
Mt1-4/MmpL5	HPU_10541	matrix metalloproteinase 14, membrane metalloproteinase
Mt1-4/MmpL6	HPU_10542	matrix metalloproteinase 17, MT4-mmp
Mt1-4/MmpL7	HPU_10538	matrix metalloproteinase 16, membrane type-matrix metalloproteinase 3
Mt5/MmpL2	HPU_10539	matrix metalloproteinase 24
Nfkbil1L	HPU_00325	nuclear factor of kappa light polypeptide gene enhancer in B-cells inhibitor-like 1-like
Npnt	HPU_02856	nephronectin
Otop2L	HPU_08382	otopetrin 2-like
P16	HPU_02490	none
Pks2	HPU_12246	none
Plod2	HPU_20953	procollagen-lysine, 2-oxoglutarate 5-dioxygenase 2; lysyl hydroxylase isoform 2
Pm27	HPU_06864	PM27
Pres_1	HPU_08584	Prestin (Solute carrier family 26 member 5)
Presnln	HPU_18198	none
Prss12L_2	HPU_14681	protease, serine, 12 (neurotrypsin, motopsin)-2; neurotrypsin-like-2
Ptpriz	HPU_23146	Fmi, Protein Tyrosine phosphatase receptor type
Sdccag3L	HPU_21187	serologically defined colon cancer antigen 3-like
Sepn1	HPU_14477	selenoprotein N, 1
Sm30A	HPU_12534	none
Sm50	HPU_11658	none
Spsb3	HPU_22355	splA/ryanodine receptor domain and SOCS box containing 3; SPRY domain and SOCS box containing 3
Timp3b	HPU_08694	tissue inhibitor of metalloproteinases
Ttrspn_19	HPU_14013	none
Ttrspn_6	HPU_23102	none
Txndc	HPU_01515	Thioredoxin domain containing protein 1 precursor (Transmembrane Trx-related protein) (Thioredoxin-related transmembrane protein)
p58-a	HPU_22824	none
p58-b	HPU_22829	none
NSM		
Abcc5D	HPU_11152	MRP5
Abcg12	HPU_05782	none
Acl	HPU_00327	ATP citrate-lyase
Acly_2	HPU_00328	ATP-citrate synthase (ATP-citrate (pro-S-)-lyase) (Citrate cleavage enzyme)
Acnn1	HPU_22805	Amiloride-sensitive cation channel 1, neuronal (Amiloride-sensitive brain sodium channel) (Brain sodium channel 1) (BNaC1)(Amiloride-sensitive cation channel neuronal 1) (BNC1) (Degenerin channel MDEG)
Ars_3	HPU_20700	Arylsulfatase precursor (AS) (Aryl-sulfate sulphohydrolase) (ARS)
B3galt1_16	HPU_18297	Beta-1,3-galactosyltransferase 1 (Beta-1,3-GalTase 1) (Beta3GalT1) (UDP-galactose:beta-N-acetyl-glucosamine-beta-1,3-galactosyltransferase 1) (UDP-Gal:betaGlcNAc beta 1,3-galactosyltranferase-I)
C20orf3_1	HPU_16418	chromosome 20 open reading frame 3 - duplicate
CcLta1-like	HPU_01999	none
Chrna10	HPU_09231	cholinergic receptor, nicotinic, alpha 10
Chrna9L_92	HPU_20127	cholinergic receptor, nicotinic, alpha 9-like-2
Clect_8	HPU_25040	none
Cpt1a	HPU_03166	Carnitine O-palmitoyltransferase I, mitochondrial liver isoform (CPT I) (CPTI-L) (Carnitine palmitoyltransferase 1A)
Css2	HPU_05179	Chondroitin sulfate synthase 2 (Glucuronosyl-N-acetylgalactosaminyl-proteoglycan 4-beta-N-acetylgalactosaminyltransferase II) (N-acetylgalactosaminyl-proteoglycan 3-beta-glucuronosyltransferase II) (Chondroitin glucuronyltransferase II) (N-acetylgalactosaminyltransferase) (Chondroitin polymerizing factor)
Cycs	HPU_10412	Cytochrome c
DagL-like	HPU_06833	Dystroglycan like
Dpgm	HPU_05682	phosphoglycerate mutase, dependent type
ElavL	HPU_19698	embryonic lethal, abnormal vision
Enpp7-2	HPU_20380	ectonucleotide pyrophosphatase/phosphodiesterase 7-2
Fah	HPU_05105	Fumarylacetoacetase (Fumarylacetoacetate hydrolase) (Beta-diketonase) (FAA)
Fmo3	HPU_04909	Dimethylaniline monooxygenase [N-oxide-forming] 3 (Hepatic flavin-containing monooxygenase 3) (FMO 3) (Dimethylaniline oxidase 3)
Fmo5_1	HPU_05784	Dimethylaniline monooxygenase [N-oxide-forming] 5 (Hepatic flavin-containing monooxygenase 5) (FMO 5) (Dimethylaniline oxidase 5)
Gale	HPU_13070	UDP-galactose-4-epimerase
Galnt7-2	HPU_01795	UDP-N-acetyl-alpha-D-galactosamine:polypeptide N-acetylgalactosaminyltransferase 7-2
Hadhb	HPU_21682	Trifunctional enzyme beta subunit, mitochondrial precursor (TP-beta) [Includes: 3-ketoacyl-CoA thiolase (Acetyl-CoA acyltransferase) (Beta-ketothiolase)]
Htpalpha	HPU_19463	enoyl-CoA hydratase/3-hydroxyacyl-CoA dehydrogenase alpha-subunit of trifunctional protein
Hypp_0119	HPU_00461	hypothetical protein-2404
Hypp_0851	HPU_03096	hypothetical protein-2515
Hypp_1512	HPU_05452	none
Hypp_1961	HPU_07108	hypothetical protein-2120; superoxide dismutase-like
Hypp_2038	HPU_07364	hypothetical protein-2798
Hypp_5484	HPU_18921	hypothetical protein-2259
Hypp_5738	HPU_19802	none
Hypp_6378	HPU_21940	hypothetical protein-1597; serine/threonine protein phosphatase-like
Hypp_6468	HPU_22276	hypothetical protein-795
Hypp_7091	HPU_24149	none
Lactb2	HPU_17951	lactamase, beta 2 protein
Mif7	HPU_07188	none
Ncag1	HPU_18960	none
Npffr2L_4	HPU_17155	neuropeptide FF receptor 2-like-4
Papss	HPU_05457	3'phosphoadenosine 5'phosphosulfate synthase, PAPS synthase, PAPS syntetase
Pc	HPU_01510	Pyruvate carboxylase, mitochondrial precursor (Pyruvic carboxylase) (PCB)
Pks1	HPU_11477	none
Pofut1-like	HPU_08250	protein-O-fucosyltransferase 1, OFUT1, OFUCT1
Slc10a2L_3	HPU_12602	solute carrier family 10, member 2-like-3
Slc25a10	HPU_16742	Mitochondrial dicarboxylate carrier
Slc26a11_1	HPU_06042	anion exchanger
Slc35f3L	HPU_20561	solute carrier family 35, member f3 or f2
Slc45a2_1-like	HPU_11514	Membrane-associated transporter protein
Spsb3	HPU_22355	splA/ryanodine receptor domain and SOCS box containing 3; SPRY domain and SOCS box containing 3
Sr/Fu/Igr	HPU_21422	none
Srcr42	HPU_14570	none
Tmf1L	HPU_19395	TATA element modulatory factor 1-like
Veg1_2_endoderm		
Bhmt_1	HPU_10406	Betaine--homocysteine S-methyltransferase)
Cadherin_6	HPU_07541	none
Cav1	HPU_03954	none
Dmgdh	HPU_10409	Dimethylglycine dehydrogenase, mitochondrial precursor (ME2GLYDH)
Fibulin	HPU_03771	fibulin-2
Hypp_0973	HPU_03634	none
Hypp_2210	HPU_07972	none
Hypp_5175	HPU_17889	hypothetical protein-1246
Hypp_5176	HPU_17891	hypothetical protein-1246
Hypp_5345	HPU_18447	none
Hypp_7295	HPU_24828	hypothetical protein-1851; aminotransferase-like
MyXII_1	HPU_22531	Sp-Myosin XII
Nvd	HPU_04111	Rieske-domain protein Neverland [Bombyx mori]; neverland
Odz3	HPU_12253	odz, odd Oz/ten-m homolog 3 (Drosophila); teneurin-3; tenascin-M3
PppL_224	HPU_03117	pol polyprotein like-224
Scube	HPU_20921	signal peptide,CUB domain,EGF-like/CEGP
Timp1b	HPU_11236	tissue inhibitor of metalloproteases
Uck2	HPU_09154	Uridine-cytidine kinase 2 (UCK 2) (Uridine monophosphokinase 2) (Cytidine monophosphokinase 2)
Apical_ectoderm		
C3orf20L	HPU_19992	human chromosome 3 open reading frame 20-like
C3orf21_1-like	HPU_14834	human chromosome 3 open reading frame 21-1
Cry1/2L	HPU_21139	Cryptochrome 1, Photolyase-like
Dynlc2-3c-like	HPU_20964	none
Dyrk4	HPU_24032	dual specificity tyrosine phosphorylation regulated kinase 4
Hypp_1056	HPU_03943	hypothetical protein-2760
Hypp_1333	HPU_04823	hypothetical protein-1273
Hypp_1641	HPU_05934	hypothetical protein-2858
Hypp_1863	HPU_06756	none
Hypp_2822	HPU_09988	none
Hypp_3903	HPU_13690	hypothetical protein-3009
Hypp_4706	HPU_16355	hypothetical protein-2880
Hypp_5844	HPU_20174	none
Hypp_7302	HPU_24855	none
Non/MmyIIhcph	HPU_19542	myosin II
Opn5L	HPU_23194	opsin 5-like
RapL	HPU_10986	rap gtpase-activating protein (mosquito)-like
Sat2_3	HPU_17265	Diamine acetyltransferase 2 (Spermidine/spermine N(1)-acetyltransferase 2) (Polyamine N-acetyltransferase 2)
Sfrp1/5	HPU_03036	none
Unk_85-like	HPU_21245	none
Wdr49	HPU_10202	WD repeat domain 49
