# Supplementary material for: Early expression onset of tissue-specific effector genes during the specification process in sea urchin embryos
Source: EvoDevo. 2023 Apr 26;14:7. doi: 10.1186/s13227-023-00210-2 (PMC10131483; doi:10.1186/s13227-023-00210-2)
Supplement: Supplementary file 1 — Additional file 1: Fig S1. Identification of cell clusters in the single-cell transcriptomic data. Fig S2. Distribution of averaged expression levels of the candidate cohort of tissue-specific effector genes in each cell cluster. Fig S3. Spatial expression pattern of the tissue-specific effector genes whose expression was estimated to be restricted to a single cell cluster. Fig S4. Temporal expression pattern of the tissue-specific effector genes whose expression was observed at 0 hpf in the representative cell lineages. Table S1. List of screened tissue-specific effector genes. Table S2. List of marker tissue-specific effector genes. [file 13227_2023_210_MOESM1_ESM.zip › 13227_2023_210_MOESM1_ESM/supplement/Table S3.pdf]

Type	Gene	Expression	Hp time	Hp stage	Known onset	Species	Reference
Effector	Pkd2	Apical_ecto, Germ_line, Non_apical_ecto, Veg1_2_endo, Veg1_ecto	12	EB-HB	EG	Pl	Tisler et al., 2016
	Netrin1	Apical_ecto, Germ_line, NSM, Non_apical_ecto, Veg1_2_endo	18	MB	MB	Lv	Slota et al., 2019
	Nan2	Germ_line	6	EB	HB	Hp	Fujii et al., 2009
	Abcc5D	NSM	0	Maternal	HB	Sp	Gökirmak et al., 2016
	Abcg12	NSM	16	HB-MB	HB	Sp	Shipp and Hamdoun, 2012
	ElavL	NSM	0	Maternal	BL	Sp	Rafiq et al., 2012
	Fmo3	NSM	16	HB-MB	HB	Sp	Calestani et al., 2003
	Galnt7-2	NSM	0	Maternal	HB	Sp	Famiglietti et al., 2017
	Pks1	NSM	16	HB-MB	HB	Sp	Calestani et al., 2003
	Adam/Ts2/3L2_1	NSM, Skeletogenic	22	MB-EG	MB	Sp	Rise and Burke, 2002
	B3galt1_16	NSM, Skeletogenic	18	MB	EB-HB	Sp	Rafiq et al., 2014
	Aqp9	Skeletogenic	20	MB	EB-HB	Sp	Rafiq et al., 2014
	C-lectin/PMC1	Skeletogenic	18	MB	MB	Sp	Rafiq et al., 2012
	C2gnt3	Skeletogenic	18	MB	EB-HB	Sp	Rafiq et al., 2014
	Enpep_2	Skeletogenic	10	EB	MB	Sp	Rafiq et al., 2014
	Fam20c	Skeletogenic	0	Maternal	MB	Sp	Rafiq et al., 2014
	Gabrb3L	Skeletogenic	24	MB-EG	MB	Sp	Rafiq et al., 2014
	Msp130	Skeletogenic	10	EB	BL	Sp	Harkey et al., 1992
	Mt1-4/MmpL5	Skeletogenic	14	HB	BL	Pl	Ragusa et al., 2017
	Mt1-4/MmpL6	Skeletogenic	14	HB	EB-HB	Sp	Rafiq et al., 2014
	Mt1-4/MmpL7	Skeletogenic	24	EG	BL	Pl	Ragusa et al., 2017
	Mt5/MmpL2	Skeletogenic	18	MB	MB	Sp	Rafiq et al., 2014
	Npnt	Skeletogenic	14	HB	EB-HB	Sp	Rafiq et al., 2014
	Otop2L	Skeletogenic	22	MB-EG	MB	Sp	Rafiq et al., 2014
	P16	Skeletogenic	16	HB-MB	LB	Lv	Cheers and Ettensohn, 2005
	p58-a	Skeletogenic	14	HB	HB	Sp	Adomako-Ankomah et al., 2011
	p58-b	Skeletogenic	12	EB-HB	HB	Sp	Adomako-Ankomah et al., 2011
	Pks2	Skeletogenic	12	EB-HB	BL	Sp	Beeble and Calestani 2012
	Pla2g1b_8	Skeletogenic	26	EG	MB	Sp	Rafiq et al., 2012
	Pm27	Skeletogenic	16	HB-MB	BL	Sp	Herkey et al., 1995
	Sm29	Skeletogenic	14	HB	BL	Sp	Rafiq et al., 2012
	Sm30A	Skeletogenic	10	EB	G	Sp	George et al., 1991, Killian et al., 2010
	Sm50	Skeletogenic	10	EB	MB	Sp	George et al., 1991
	Bhmt_1	Veg1_2_endoderm	0	Maternal	HB	Pl	Röttinger et al., 2008
TF	Ac/Sc	Apical_ectoderm	18	MB	MB	Sp	Burke et al., 2006
	FoxQ2_1	Apical_ectoderm	6	EB	Cleavage	Sp	Yaguchi et al., 2008
	Hbn	Apical_ectoderm	10	EB	EB	Hp	Yaguchi et al., 2016
	Nkx2.1	Apical_ectoderm	14	HB	HB	Sp	Takacs et al., 2004
	Nkx3-2	Apical_ectoderm	16	HB-MB	MB	Sp	Wei et al., 2011
	Six3	Apical_ectoderm	8	EB	Cleavage	Sp	Yaguchi et al., 2007
	Z133_1	Apical_ectoderm	16	HB-MB	MB	Sp	Feuda and Peter 2022
	Soxb1	Apical_ectoderm, NSM, Veg1_2_endoderm	0	Maternal	Maternal	Sp	Kenny et al., 1999
	Ese	NSM	8	EB	MB	Sp	Rizzo et al., 2006
	GataC	NSM	16	HB-MB	HB	Sp	Materna et al., 2013
	GataL	NSM	12	EB-HB	HB	Sp	Ben-Tabou de-Leon, 2016
	Not	NSM	8	EB	MB	Sp	Peterson et al., 1999
	Runt1-like	NSM	16	HB-MB	Cleavage	Sp	Coffman et al., 1996
	Scl	NSM	0	Maternal	Maternal	Sp	Materna et al., 2013
	Six1/2	NSM	10	EB	MB	Sp	Ben-Tabou de-Leon, 2016
	Z166	NSM	0	Maternal	HB	Sp	Materna et al., 2013
	Bra	NSM, Veg1_2_endoderm	14	HB	EB	Pl	Croce et al., 2001
	Eve	NSM, Veg1_2_endoderm	6	EB	Cleavage	Sp	Ransick et al., 2002
	FoxA	NSM, Veg1_2_endoderm	8	EB	EB	Sp	Oliveri et al., 2006
	Gcm	NSM, Veg1_2_endoderm	10	EB	EB	Sp	Ransick and Davidson, 2006
	Hox11/13b	NSM, Veg1_2_endoderm	0	Maternal	EB	Sp	Cui et al., 2017
	Krl	NSM, Veg1_2_endoderm	6	EB	Cleavage	Hp	Yamazaki et al., 2008
	Notch	NSM, Veg1_2_endoderm	0	Maternal	Maternal	Lv	Sherwood and McClay, 1997
	Delta	Skeletogenic	8	EB	EB	Sp	Materna et al., 2010
	Dr_1	Skeletogenic	12	EB-HB	EB	Sp	Amore et al., 2003
	Ets1/2	Skeletogenic	0	Maternal	Maternal	Sp	Rizzo et al., 2006
	FoxB	Skeletogenic	20	MB	MB	Sp	Minokawa et al., 2004
	FoxO_1	Skeletogenic	20	MB	MB	Sp	Tu et al., 2006
	Hnf6	Skeletogenic	0	Maternal	Maternal	Sp	Otim et al., 2004
	Mitf	Skeletogenic	16	HB-MB	Maternal	Pl	Russo et al., 2019
	Pdgfr/vegfrL	Skeletogenic	18	MB	MB	Pl	Duloquin et al., 2007
	Pmar1a	Skeletogenic	6	EB	Cleavage	Sp	Oliveri et al., 2003
	Tbr	Skeletogenic	0	Maternal	EB	Pl	Croce et al., 2001
	Tel	Skeletogenic	0	Maternal	Maternal	Sp	Rizzo et al., 2006
	Erg	Skeletogenic, NSM	14	HB	HB	Sp	Rizzo et al., 2006
	Hhex	Skeletogenic, NSM	10	EB	MB	Sp	Howard-Ashby et al., 2006
	Blimp1	Skeletogenic, NSM, Veg1_2_endoderm	0	Maternal	MB	Sp	Livi and Davidson, 2006
	Wnt8	Skeletogenic, NSM, Veg1_2_endoderm	6	EB	Cleavage	Sp	Range et al., 2013
	Tgif	Skeletogenic, Veg1_2_endoderm	0	Maternal	Maternal	Sp	Howard-Ashby et al., 2006
	Hh	Veg1_2_endoderm	30	EG	EG	Lv	Walton et al., 2009
	Lim1	Veg1_2_endoderm	0	Maternal	Maternal	Hp	Kawasaki et al., 1999
	Wnt16	Veg1_2_endoderm	0	Maternal	Maternal	Sp	Martínez-Bartolomé and Range, 2019
