# Supplementary material for: Early expression onset of tissue-specific effector genes during the specification process in sea urchin embryos
Source: EvoDevo. 2023 Apr 26;14:7. doi: 10.1186/s13227-023-00210-2 (PMC10131483; doi:10.1186/s13227-023-00210-2)
Supplement: Supplementary file 1 — Additional file 1: Fig S1. Identification of cell clusters in the single-cell transcriptomic data. Fig S2. Distribution of averaged expression levels of the candidate cohort of tissue-specific effector genes in each cell cluster. Fig S3. Spatial expression pattern of the tissue-specific effector genes whose expression was estimated to be restricted to a single cell cluster. Fig S4. Temporal expression pattern of the tissue-specific effector genes whose expression was observed at 0 hpf in the representative cell lineages. Table S1. List of screened tissue-specific effector genes. Table S2. List of marker tissue-specific effector genes. [file Table S1.pdf › 13227_2023_210_MOESM1_ESM/supplement/Table S1.pdf]

Table S1: Gene list of screened tissue-specific effector genes

#	Gene(Hp id)	Cell lineages	Uniprot annotation
1	Abcc10a(HPU_18834)	Apical_ecto	MRP7
1	C15orf33L(HPU_00107)	Apical_ecto	human chromosome 15 open reading frame 33-like
1	CcLta1.2(HPU_00775)	Apical_ecto	none
1	Fn3/Egff_7-like(HPU_13269)	Apical_ecto	none
1	Hypp_0012(HPU_00067)	Apical_ecto	hypothetical protein-1499; protein kinase-like
1	Hypp_1056(HPU_03943)	Apical_ecto	hypothetical protein-2760
1	Hypp_1641(HPU_05934)	Apical_ecto	hypothetical protein-2858
1	Hypp_2064(HPU_07470)	Apical_ecto	hypothetical protein-2442; tolloid-like
1	Hypp_2073(HPU_07496)	Apical_ecto	none
1	Hypp_2822(HPU_09988)	Apical_ecto	none
1	Hypp_4706(HPU_16355)	Apical_ecto	hypothetical protein-2880
1	Kiaa1529L_3(HPU_03500)	Apical_ecto	KIAA1529 protein-like-3
1	Mab21l2(HPU_01150)	Apical_ecto	mab-21-like 2 (C. elegans)
1	Nlr89-like(HPU_12370)	Apical_ecto	NACHT and LRR containing protein
1	RapL(HPU_10986)	Apical_ecto	rap gtpase-activating protein (mosquito)-like
1	Abp34L(HPU_14243)	Germ_line	alpha- and gamma-adaptin-binding protein p34-like
1	C10orf4(HPU_21110)	Germ_line	human chromosome 10 open reading frame 4
1	Dnl4(HPU_03463)	Germ_line	ligase IV, DNA, ATP-dependent
1	EhH4_79(HPU_18775)	Germ_line	none
1	Erk3/4(HPU_16119)	Germ_line	mitogen activated protein kinase 6, ERK-3/4, p97 ERK
1	Hypp_2071(HPU_07490)	Germ_line	none
1	Hypp_2236(HPU_08044)	Germ_line	hypothetical protein-2494
1	Hypp_3021(HPU_10672)	Germ_line	hypothetical protein-2012
1	Hypp_4050(HPU_14163)	Germ_line	hypothetical protein-110
1	Hypp_5888(HPU_20300)	Germ_line	none
1	Hypp_6595(HPU_22676)	Germ_line	hypothetical protein-205; KIAA0556-like
1	Lhh2be(HPU_23641)	Germ_line	none
1	Mgc16943L(HPU_24741)	Germ_line	hypothetical protein LOC112479
1	Mpp6(HPU_11838)	Germ_line	membrane protein, palmitoylated 6 (or 2 or 3) (MAGUK p55 subfamily, member 6)
1	Nan2(HPU_04462)	Germ_line	none
1	Ndufaf1(HPU_03893)	Germ_line	NADH dehydrogenase (ubiquinone) 1 alpha subcomplex assembly factor 1
1	Parvb(HPU_24873)	Germ_line	parvin, beta; affixin
1	Pdha1(HPU_13329)	Germ_line	Pyruvate dehydrogenase E1 component alpha subunit, somatic form, mitochondrial precursor (PDHE1-A type I)
1	Rwdd1(HPU_05200)	Germ_line	RWD domain containing 1
1	Smc6-2(HPU_18878)	Germ_line	structural maintenance of chromosomes 6-2; SMC6 protein
1	Stx16(HPU_17590)	Germ_line	syntaxin 16
1	Taf5(HPU_23409)	Germ_line	TBP-associated factor 5
1	Tbpaf_rna_polIB(HPU_20372)	Germ_line	SpTAF1B
1	Tho1L(HPU_10317)	Germ_line	THO complex 1, Nuclear matrix
1	Tmem48(HPU_09554)	Germ_line	transmembrane protein 48
1	Tmps9-like(HPU_16694)	Germ_line	secreted trypsin-like serine protease
1	6Afcol(HPU_20868)	NSM	none
1	A2col(HPU_10208)	NSM	none
1	Aasdhppt(HPU_13523)	NSM	Aminoadipate-semialdehyde dehydrogenase-phosphopantetheinyl transferase
1	Abcc5A(HPU_18843)	NSM	MRP5, MOAT
1	Abcg12(HPU_05782)	NSM	none
1	Acaca(HPU_21302)	NSM	Acetyl-CoA carboxylase (ACC) [Includes: Biotin carboxylase] (acetyl-Coenzyme A carboxylase alpha)
1	Acap2_1(HPU_16524)	NSM	ArfGAP with coiled-coil, ankyrin repeat and PH domains 2-1; centaurin, beta 2-1
1	Acl(HPU_00327)	NSM	ATP citrate-lyase
1	Acnn1(HPU_22805)	NSM	Amiloride-sensitive cation channel 1, neuronal (Amiloride-sensitive brain sodium channel) (Brain sodium channel 1) (BNaC1)(Amiloride-sensitive cation channel neuronal 1) (BNC1) (Degenerin channel MDEG)
1	Apc-like(HPU_16482)	NSM	none
1	Arl11(HPU_14222)	NSM	ADP-Ribosylation Factor-Like 11
1	Atub3(HPU_18940)	NSM	none
1	C20orf3_1(HPU_16418)	NSM	chromosome 20 open reading frame 3 - duplicate
1	Calm2-like(HPU_00171)	NSM	calmodulin 2 (phosphorylase kinase, delta)
1	Chrna10(HPU_09231)	NSM	cholinergic receptor, nicotinic, alpha 10
1	Clect/Tm_3-like(HPU_11610)	NSM	none
1	Clect_112-like(HPU_03641)	NSM	none
1	Cryz(HPU_17959)	NSM	Quinone oxidoreductase (NADPH:quinone reductase) (Zeta-crystallin)
1	Dhyg_2(HPU_13626)	NSM	short chain dehydrogenase family
1	Dysf(HPU_08020)	NSM	dysferlin (various isoforms)
1	Endrvt8(HPU_14291)	NSM	endonuclease/reverse transcriptase-like-8
1	Fat1L-like(HPU_24771)	NSM	FAT tumor suppressor homolog 1 (Drosophila)-like
1	Fh1(HPU_03075)	NSM	Fumarate hydratase 1
1	Fmo5_1(HPU_05784)	NSM	Dimethylaniline monooxygenase [N-oxide-forming] 5 (Hepatic flavin-containing monooxygenase 5) (FMO 5) (Dimethylaniline oxidase 5)
1	Gal3st3L_2(HPU_10405)	NSM	galactose-3-O-sulfotransferase 3-like-2
1	Got1(HPU_15439)	NSM	aspartate aminotransferase 1; glutamic-oxaloacetic transaminase 1, soluble
1	Gpr54L_3(HPU_11151)	NSM	G protein-coupled receptor 54-like-3
1	Hpd_1(HPU_06170)	NSM	4-hydroxyphenylpyruvate dioxygenase (4HPPD) (HPD) (HPPDase)
1	Hrh3L_19(HPU_12484)	NSM	histamine receptor H3-like-19
1	Hypp_0160(HPU_00576)	NSM	hypothetical protein-1407; GLI pathogenesis-related 1 like
1	Hypp_0674(HPU_02480)	NSM	hypothetical protein-1087; M-phase phosphoprotein 9-like
1	Hypp_0836(HPU_03026)	NSM	hypothetical protein-1249
1	Hypp_0993(HPU_03701)	NSM	none
1	Hypp_1051(HPU_03934)	NSM	none
1	Hypp_1351(HPU_04889)	NSM	hypothetical protein-721
1	Hypp_1673(HPU_06069)	NSM	hypothetical protein-1617
1	Hypp_2968(HPU_10473)	NSM	hypothetical protein-1433; G protein-coupled receptor
1	Hypp_2994(HPU_10557)	NSM	none
1	Hypp_3990(HPU_13954)	NSM	Hypothetical protein_3007
1	Hypp_3993(HPU_13960)	NSM	none
1	Hypp_4113(HPU_14402)	NSM	hypothetical protein-532
1	Hypp_4417(HPU_15423)	NSM	none
1	Hypp_4689(HPU_16281)	NSM	hypothetical protein-464
1	Hypp_5288(HPU_18259)	NSM	none
1	Hypp_6239(HPU_21482)	NSM	hypothetical protein-2100; fatty acid binding protein 1, liver-like
1	Hypp_6504(HPU_22397)	NSM	none
1	Iqwd1-like(HPU_04738)	NSM	IQ motif and WD repeats 1
1	Irf4(HPU_04538)	NSM	none
1	Kiaa0329(HPU_12758)	NSM	KIAA0329 protein
1	Kirrel2L_1(HPU_18168)	NSM	kin of IRRE like 2 (Drosophila)-like-1
1	Lrp4-3(HPU_22176)	NSM	low density lipoprotein receptor-related protein 4-3
1	Mtx2(HPU_00031)	NSM	metaxin 2
1	Nme1-like(HPU_13950)	NSM	Nucleoside diphosphate kinase NBR-A (NDK NBR-A) (NDP kinase NBR-A)
1	Npffr2L_4(HPU_17155)	NSM	neuropeptide FF receptor 2-like-4
1	Pgls(HPU_08602)	NSM	6-phosphogluconolactonase (6PGL)
1	Pgm1(HPU_04226)	NSM	phosphoglutamase 1
1	Prp19-like(HPU_04199)	NSM	PRP19 pre-mRNA processing factor 19 homolog
1	Ron(HPU_08565)	NSM	c-met, c-sea, ron, msp receptor
1	Rptpd_fn3r-like(HPU_24614)	NSM	PTPRD, Receptor-type tyrosine-protein phosphatase delta precursor, Protein-tyrosine phosphatase delta
1	Sacm1L(HPU_15190)	NSM	SAC1 suppressor of actin mutation 1-like (yeast)
1	Sarmr6(HPU_23228)	NSM	Sp-Sarm-related 6
1	Sdr(HPU_08967)	NSM	short-chain dehydrogenease/reductase
1	Sea/Hyr/Clect(HPU_02410)	NSM	none
1	Selenbp1_1(HPU_06540)	NSM	selenium binding protein 1
1	Slc10a2L_3(HPU_12602)	NSM	solute carrier family 10, member 2-like-3
1	Slc25a27_1(HPU_07814)	NSM	Mitochondrial uncoupling protein 4 (UCP 4) (Solute carrier family 25, member 27)
1	Slc26a5(HPU_10192)	NSM	solute carrier family 26, member 5, prestin
1	Slc36a1_2(HPU_07418)	NSM	Proton-coupled amino acid transporter 1 (Proton/amino acid transporter 1) (Solute carrier family 36 member 1)
1	Slc46a3_1(HPU_08322)	NSM	solute carrier family 46, member 3-1
1	Slco4a1_14-like(HPU_07227)	NSM	Solute carrier organic anion transporter family member 4A1 (Solute carrier family 21 member 12) (Sodium-independent organic anion transporter E) (Organic anion transporting polypeptide E) (OATP-E)
1	Srcr152(HPU_11935)	NSM	none
1	Srcr68(HPU_11934)	NSM	none
1	Surreal/Gpcr109(HPU_13993)	NSM	none
1	Tk11(HPU_20757)	NSM	tyrosine kinase receptor
1	Trpc6(HPU_11475)	NSM	Short transient receptor potential channel 6 (TrpC6)
1	Ucp3(HPU_06309)	NSM	Mitochondrial uncoupling protein 3 (UCP 3)
1	Ugt8_2(HPU_01021)	NSM	2-hydroxyacylsphingosine 1-beta-galactosyltransferase precursor (UDP-galactose-ceramide galactosyltransferase) (Ceramide UDP-galactosyltransferase) (Cerebroside synthase)
1	Unk_78(HPU_09212)	NSM	unknown kinase
1	Wdr25(HPU_00912)	NSM	WD repeat domain 25
1	Frs3L-like(HPU_17236)	Non_apical_ecto	fibroblast growth factor receptor substrate 3-like
1	Hypp_0607(HPU_02253)	Non_apical_ecto	none
1	Hypp_1504(HPU_05430)	Non_apical_ecto	none
1	Hypp_3538(HPU_12470)	Non_apical_ecto	none
1	Hypp_4130(HPU_14443)	Non_apical_ecto	hypothetical protein-930
1	Hypp_5064(HPU_17578)	Non_apical_ecto	none
1	Hypp_5708(HPU_19720)	Non_apical_ecto	none
1	Lhh2bj(HPU_07434)	Non_apical_ecto	none
1	Med13l(HPU_07159)	Non_apical_ecto	mediator complex subunit 13-like; mdiator of RNA polymerase II transcription subunit-like
1	Mmp21L(HPU_07346)	Non_apical_ecto	matrix metalloproteinase 21
1	Polg(HPU_21865)	Non_apical_ecto	DNA polymerase gamma
1	Rergl2(HPU_14200)	Non_apical_ecto	Ras-like estrogen-regulated, growth inhibitor Like 2
1	TrafB(HPU_18838)	Non_apical_ecto	none
1	TrypL(HPU_12515)	Non_apical_ecto	trypsin-like serine protease with CCP/sushi repeats
1	Unc44L_103(HPU_13709)	Non_apical_ecto	ankyrin2,3/unc44-like-103
1	Aji(HPU_01160)	Skeletogenic	none
1	Aqp9(HPU_16557)	Skeletogenic	Aquaporin 9 (Small solute channel 1)
1	Arl11l2(HPU_14221)	Skeletogenic	ARL11-Like 2, ADP-Ribosylation Factor-Like 11 LIKE 2
1	B4galt6-like(HPU_07694)	Skeletogenic	Beta-1,4-galactosyltransferase 6 (Beta-1,4-GalTase 6) (Beta4Gal-T6) (b4Gal-T6) (UDP-galactose:beta-N-acetylglucosamine beta-1,4-galactosyltransferase 6) (UDP-Gal:beta-GlcNAc beta-1,4-galactosyltransferase 6) [Includes: Lactosylceramide synthase (LacCer synthase) (UDP-Gal:glucosylceramide beta-1,4-galactosyltransferase)]
1	C-lectin(HPU_21887)	Skeletogenic	none
1	C-lectin/PMC1(HPU_05420)	Skeletogenic	none
1	C2gnt3(HPU_11202)	Skeletogenic	core 2 beta-1,6-N-acetylglucosaminyltransferase 3
1	Cara7LA(HPU_10850)	Skeletogenic	none
1	Cept1(HPU_15538)	Skeletogenic	Choline/ethanolaminephosphotransferase 1
1	Clect_13(HPU_23164)	Skeletogenic	none
1	Clect_25(HPU_06862)	Skeletogenic	none
1	Clect_76(HPU_15310)	Skeletogenic	none
1	Dync2h1-like(HPU_16545)	Skeletogenic	dynein, cytoplasmic 2, heavy chain 1
1	Enpep_2(HPU_10871)	Skeletogenic	Glutamyl aminopeptidase (EAP) (Aminopeptidase A) (APA) (Differentiation antigen gp160)
1	Exoc6(HPU_08228)	Skeletogenic	exocyst complex component 6, Sec15
1	Fam55c(HPU_16812)	Skeletogenic	family with sequence similarity 55, member C
1	Fam55cL_2(HPU_08964)	Skeletogenic	family with sequence similarity 55, member C-like-2
1	Fn3/Egff_1(HPU_05856)	Skeletogenic	none
1	Gabrb3L(HPU_06446)	Skeletogenic	gamma-aminobutyric acid (GABA) A receptor, beta 3-like
1	Galnt1(HPU_02901)	Skeletogenic	UDP-N-acetyl-alpha-D-galactosamine:polypeptide N-acetylgalactosaminyltransferase 1 (GalNAc-T1); polypeptide GalNAc transferase 1
1	Gcnt1(HPU_12533)	Skeletogenic	glucosaminyl (N-acetyl) transferase 1, core 2
1	Gns(HPU_06431)	Skeletogenic	N-acetylglucosamine-6-sulfatase precursor (G6S) (Glucosamine-6-sulfatase)
1	Hypp_0677(HPU_02487)	Skeletogenic	none
1	Hypp_0678(HPU_02488)	Skeletogenic	hypothetical protein-347; G protein-coupled receptor-like
1	Hypp_0679(HPU_02489)	Skeletogenic	hypothetical protein-2386
1	Hypp_0681(HPU_02493)	Skeletogenic	hypothetical protein-2999
1	Hypp_1780(HPU_06417)	Skeletogenic	hypothetical protein-2174; inversin-like
1	Hypp_1811(HPU_06530)	Skeletogenic	none
1	Hypp_1858(HPU_06741)	Skeletogenic	hypothetical protein-2712
1	Hypp_2991(HPU_10550)	Skeletogenic	hypp-120
1	Hypp_3399(HPU_12019)	Skeletogenic	none
1	Hypp_4134(HPU_14470)	Skeletogenic	hypothetical protein-3018
1	Hypp_4432(HPU_15488)	Skeletogenic	hypothetical protein-740
1	Hypp_4562(HPU_15890)	Skeletogenic	none
1	Hypp_4911(HPU_17024)	Skeletogenic	none
1	Hypp_4999(HPU_17334)	Skeletogenic	none
1	Hypp_5094(HPU_17672)	Skeletogenic	none
1	Hypp_5232(HPU_18109)	Skeletogenic	hypothetical protein-2072
1	Hypp_6368(HPU_21914)	Skeletogenic	hypothetical protein-1512
1	Hypp_6369(HPU_21916)	Skeletogenic	none
1	Hypp_6370(HPU_21917)	Skeletogenic	hypothetical protein-2035
1	Hypp_6416(HPU_22074)	Skeletogenic	none
1	Hypp_6519(HPU_22431)	Skeletogenic	hypothetical protein-1159; trans-sialidase-like
1	Hypp_6651(HPU_22821)	Skeletogenic	none
1	Hypp_6785(HPU_23229)	Skeletogenic	hypothetical protein-70
1	Lrr/Fn3f(HPU_15080)	Skeletogenic	none
1	Lrr/Igr_10(HPU_11488)	Skeletogenic	none
1	Lrrc68-like(HPU_12769)	Skeletogenic	leucine rich repeat containing 68
1	Mamdc4-15-like(HPU_14506)	Skeletogenic	MAM domain containing 4-15
1	Megf6-2(HPU_21918)	Skeletogenic	multiple EGF-like-domains 6-2
1	Msp130L(HPU_05527)	Skeletogenic	mesenchyme-specific cell surface glycoprotein-like
1	Msp130r1(HPU_05532)	Skeletogenic	none
1	Mt1-4/MmpL5(HPU_10541)	Skeletogenic	matrix metalloproteinase 14, membrane metalloproteinase
1	Mt1-4/MmpL6(HPU_10542)	Skeletogenic	matrix metalloproteinase 17, MT4-mmp
1	Mt1-4/MmpL7(HPU_10538)	Skeletogenic	matrix metalloproteinase 16, membrane type-matrix metalloproteinase 3
1	Mt5/MmpL2(HPU_10539)	Skeletogenic	matrix metalloproteinase 24
1	Nfkbil1L(HPU_00325)	Skeletogenic	nuclear factor of kappa light polypeptide gene enhancer in B-cells inhibitor-like 1-like
1	Nrl(HPU_05066)	Skeletogenic	none
1	Otop2L(HPU_08382)	Skeletogenic	otopetrin 2-like
1	P16(HPU_02490)	Skeletogenic	none
1	Pla2g1b_8(HPU_24778)	Skeletogenic	Phospholipase A2 precursor (Phosphatidylcholine 2-acylhydrolase)(Group IB phospholipase A2)
1	Prss12L_2(HPU_14681)	Skeletogenic	protease, serine, 12 (neurotrypsin, motopsin)-2; neurotrypsin-like-2
1	Radil(HPU_01998)	Skeletogenic	Rap GTPase interactor; Ras-associating and dilute domain-containing protein
1	Slc6a3-like(HPU_20147)	Skeletogenic	solute carrier family 6 (neurotransmitter transporter, dopamine), member 3
1	Sm29(HPU_23163)	Skeletogenic	none
1	Spc39L-like(HPU_05152)	Skeletogenic	solute carrier family 39 (zinc transporter)-like
1	Timp3b(HPU_08694)	Skeletogenic	tissue inhibitor of metalloproteinases
1	Trpm3L(HPU_16400)	Skeletogenic	transient receptor potential cation channel, subfamily M, member 3-like
1	Aqp8(HPU_16073)	Veg1_2_endo	Aquaporin 8
1	Cadherin_6(HPU_07541)	Veg1_2_endo	none
1	Chst11(HPU_11207)	Veg1_2_endo	carbohydrate sulfotransferase 11; chondroitin 4-sulfotranseferase
1	Clca1-2-like(HPU_15996)	Veg1_2_endo	chloride channel accessory 1-2
1	Elovl4(HPU_09538)	Veg1_2_endo	Elongation of very long chain fatty acids protein 4
1	Glyphp3(HPU_23897)	Veg1_2_endo	none
1	Hypp_2469(HPU_08768)	Veg1_2_endo	none
1	Hypp_2544(HPU_09040)	Veg1_2_endo	none
1	Hypp_3146(HPU_11081)	Veg1_2_endo	none
1	Hypp_4142(HPU_14532)	Veg1_2_endo	none
1	Hypp_5175(HPU_17889)	Veg1_2_endo	hypothetical protein-1246
1	Hypp_5611(HPU_19405)	Veg1_2_endo	none
1	Itih(HPU_05232)	Veg1_2_endo	none
1	Lktnh(HPU_12103)	Veg1_2_endo	none
1	Lyss(HPU_18473)	Veg1_2_endo	lysyl-tRNA synthetase
1	Mpnd(HPU_02133)	Veg1_2_endo	MPN domain containing
1	Nos_1(HPU_17332)	Veg1_2_endo	none
1	Notum(HPU_08466)	Veg1_2_endo	Wingful, WF
1	Pwp2h(HPU_22043)	Veg1_2_endo	PWP2 periodic tryptophan protein homolog (yeast)
1	Rdh1_1(HPU_20791)	Veg1_2_endo	retinol dehydrogenase type 1
1	Rergl4(HPU_12694)	Veg1_2_endo	Ras-like estrogen-regulated, growth inhibitor like 4
1	Si_1(HPU_21318)	Veg1_2_endo	sucrase-isomaltase (alpha-glucosidase)
1	Susd2-3(HPU_00322)	Veg1_2_endo	sushi domain containing 2-3
1	Ugt2b34(HPU_11263)	Veg1_2_endo	UDP glucuronosyltransferase 2 family, polypeptide B34
1	Wscd1(HPU_22351)	Veg1_2_endo	WSC domain containing 1
1	Acot2L(HPU_14260)	Veg1_ecto	acy-coA thioesterase 2-like
1	Bmp1/TldL1(HPU_19888)	Veg1_ecto	none
1	Efcab8(HPU_18443)	Veg1_ecto	EF-hand calcium binding domain 8
1	Gluld1_6(HPU_02871)	Veg1_ecto	Glutamate--ammonia ligase domain containing 1 protein (Lengsin) (Lens glutamine synthase-like)
1	Gss_1(HPU_05800)	Veg1_ecto	Glutathione synthetase (Glutathione synthase) (GSH synthetase) (GSH-S)
1	Hypp_0114(HPU_00426)	Veg1_ecto	hypothetical protein-1508; protein tyrosine/serine phosphatase (bacterial)-like
1	Hypp_1130(HPU_04207)	Veg1_ecto	hypothetical protein-2706; euchromatic histone methyltransferase 1-like
1	Hypp_4210(HPU_14744)	Veg1_ecto	none
1	Hypp_4766(HPU_16550)	Veg1_ecto	none
1	Hypp_5866(HPU_20238)	Veg1_ecto	none
1	Lrp4L_2-like(HPU_21521)	Veg1_ecto	low density lipoprotein receptor-related protein 4-like-2
1	MacpfB3(HPU_13261)	Veg1_ecto	none
1	Pofut1_2(HPU_14210)	Veg1_ecto	protein O-fucosyltransferase 1-2
1	Slc24a5(HPU_16907)	Veg1_ecto	solute carrier family 24 (sodium/potassium/calcium exchanger), member 5
1	Slc43a3_2-like(HPU_22933)	Veg1_ecto	solute carrier family 43, member 3
1	Tmtc2(HPU_24929)	Veg1_ecto	transmembrane and tetratricopeptide repeat containing 2
1	Vwde(HPU_24901)	Veg1_ecto	von Willebrand factor D and EGF domains
2	Hypp_5795(HPU_19995)	Apical_ecto, Germ_line	none
2	Adcy3(HPU_08165)	Apical_ecto, Non_apical_ecto	Adenylate cyclase type III (Adenylate cyclase, olfactive type) (ATP pyrophosphate-lyase 3) (Adenylyl cyclase 3) (AC-III) (AC3)
2	C2orf63L(HPU_22078)	Apical_ecto, Non_apical_ecto	human chromosome 2 open reading frame 63-like
2	CttnL-like(HPU_13135)	Apical_ecto, Non_apical_ecto	cortactin-like
2	Dynlc2-3c-like(HPU_20964)	Apical_ecto, Non_apical_ecto	none
2	Hypp_1863(HPU_06756)	Apical_ecto, Non_apical_ecto	none
2	Hypp_2175(HPU_07820)	Apical_ecto, Non_apical_ecto	hypothetical protein-1421; cytochrome P450-like
2	Opn5L(HPU_23194)	Apical_ecto, Non_apical_ecto	opsin 5-like
2	Sat2_3(HPU_17265)	Apical_ecto, Non_apical_ecto	Diamine acetyltransferase 2 (Spermidine/spermine N(1)-acetyltransferase 2) (Polyamine N-acetyltransferase 2)
2	Hypp_0640(HPU_02382)	Apical_ecto, Skeletogenic	none
2	C1orf201(HPU_13071)	Apical_ecto, Veg1_ecto	human chromosome 1 open reading frame 201
2	Hypp_2226(HPU_08026)	Apical_ecto, Veg1_ecto	none
2	Hypp_4331(HPU_15138)	Apical_ecto, Veg1_ecto	none
2	Morn1(HPU_15362)	Apical_ecto, Veg1_ecto	Morn domain containing
2	Glyt_3(HPU_13796)	Germ_line, NSM	glycine transporter
2	Gpr175(HPU_16505)	Germ_line, NSM	G protein-coupled receptor 175
2	Deadbpuknl-like(HPU_15095)	Germ_line, Non_apical_ecto	U5 small nuclear ribonucleoprotein 200 kDa helicase (U5 snRNP-specific 200 kDa protein) (U5-200KD) (Activating signal cointegrator 1 complex subunit 3-like 1) or U5 snRNP-specific 200 kDa protein
2	Hypp_4445(HPU_15525)	Germ_line, Non_apical_ecto	hypothetical protein-1248; human chromosome 8 open reading frame 48-like
2	RigIL3(HPU_01488)	Germ_line, Non_apical_ecto	Sp-MDA-5 like 11, Sp-LGP2 like 11
2	Pks2(HPU_12246)	Germ_line, Skeletogenic	none
2	Pres_1(HPU_08584)	Germ_line, Skeletogenic	Prestin (Solute carrier family 26 member 5)
2	Fn3f_4(HPU_06951)	Germ_line, Veg1_2_endo	none
2	Trim2L_2(HPU_01502)	Germ_line, Veg1_ecto	BAG09752.1
2	Tbc1d30(HPU_00589)	NSM, Non_apical_ecto	TBC1 domain family, member 30
2	Wsb1(HPU_12406)	NSM, Non_apical_ecto	WD repeat and SOCS box-containing 1
2	Ada_1(HPU_15661)	NSM, Skeletogenic	Adenosine deaminase (Adenosine aminohydrolase)
2	Adam/Ts2/3L2_1(HPU_18388)	NSM, Skeletogenic	none
2	Adap1L(HPU_01796)	NSM, Skeletogenic	ArfGAP with dual PH domains 1; centaurin, alpha 1-like
2	AnL8_1(HPU_16876)	NSM, Skeletogenic	astacin, tolloid-like, BMP1-like
2	Ars_3(HPU_20700)	NSM, Skeletogenic	Arylsulfatase precursor (AS) (Aryl-sulfate sulphohydrolase) (ARS)
2	B3galt1_16(HPU_18297)	NSM, Skeletogenic	Beta-1,3-galactosyltransferase 1 (Beta-1,3-GalTase 1) (Beta3GalT1) (UDP-galactose:beta-N-acetyl-glucosamine-beta-1,3-galactosyltransferase 1) (UDP-Gal:betaGlcNAc beta 1,3-galactosyltranferase-I)
2	Dcst2(HPU_10750)	NSM, Skeletogenic	DC-STAMP domain containing 2
2	Dynact1ph(HPU_02602)	NSM, Skeletogenic	dynactin 1, p150-glued
2	EceL(HPU_04055)	NSM, Skeletogenic	endothelin converting enzyme-
2	Fam167a-like(HPU_13481)	NSM, Skeletogenic	family with sequence similarity 167, member A; human chromosome 8 open reading frame 13-like
2	Fam55cL(HPU_21374)	NSM, Skeletogenic	family with sequence similarity 55, member C-like
2	Fgd4(HPU_06653)	NSM, Skeletogenic	FIVE, RhoGEF and PH domain containing protein 4, Frabin
2	Galnt7(HPU_01794)	NSM, Skeletogenic	UDP-N-acetyl-alpha-D-galactosamine:polypeptide N-acetylgalactosaminyltransferase 7
2	Gorasp2l(HPU_06337)	NSM, Skeletogenic	golgi reassembly stacking protein 2
2	Hypp_0119(HPU_00461)	NSM, Skeletogenic	hypothetical protein-2404
2	Hypp_0450(HPU_01626)	NSM, Skeletogenic	hypothetical protein-95; tetratricopeptide repeat protein; nephronophthisis 3-like; Grp94 neighboring nucleotidase-like
2	Hypp_0545(HPU_01991)	NSM, Skeletogenic	hypothetical protein-1377
2	Hypp_0851(HPU_03096)	NSM, Skeletogenic	hypothetical protein-2515
2	Hypp_1085(HPU_04041)	NSM, Skeletogenic	hypothetical protein-1908
2	Hypp_3511(HPU_12414)	NSM, Skeletogenic	hypothetical protein-395; growth arrest-specific 2-like
2	Hypp_4232(HPU_14826)	NSM, Skeletogenic	hypothetical protein-206; plexin-like
2	Hypp_4387(HPU_15343)	NSM, Skeletogenic	none
2	Hypp_5738(HPU_19802)	NSM, Skeletogenic	none
2	Hypp_6027(HPU_20753)	NSM, Skeletogenic	none
2	Kif16L-like(HPU_18306)	NSM, Skeletogenic	SNX23_HUMAN, Kinesin-like motor protein C20orf23, Sorting nexin 23
2	Map1aph(HPU_02724)	NSM, Skeletogenic	microtubule-associated protein 1A
2	Plxdc2(HPU_12889)	NSM, Skeletogenic	plexin domain containing 2; tumor endothelial marker 7-related precursor
2	Samd11(HPU_08804)	NSM, Skeletogenic	sterile alpha motif domain containing 11
2	Sarmr14(HPU_20359)	NSM, Skeletogenic	Sp-Sarm-related 14
2	Setd3(HPU_12015)	NSM, Skeletogenic	SET domain containing 3
2	Vparpf_3(HPU_00027)	NSM, Skeletogenic	none
2	p58-b(HPU_22829)	NSM, Skeletogenic	none
2	Atub2-like(HPU_06989)	NSM, Veg1_2_endo	none
2	Nav3l(HPU_05651)	NSM, Veg1_2_endo	neuron navigator 3-like
2	Nkd2(HPU_20594)	NSM, Veg1_2_endo	Naked Cuticle-2
2	C21orf57(HPU_05766)	NSM, Veg1_ecto	human chromosome 21 open reading frame 57; putative metalloprotease
2	Gpaa1(HPU_07747)	Non_apical_ecto, Skeletogenic	glycosylphosphatidylinositol anchor attachment protein 1
2	Slc16a7_1(HPU_00690)	Non_apical_ecto, Skeletogenic	Monocarboxylate transporter 2 (MCT 2)
2	Abce1(HPU_12292)	Non_apical_ecto, Veg1_2_endo	none
2	Chst1_3(HPU_12621)	Non_apical_ecto, Veg1_2_endo	carbohydrate sulfotransferase 1, keratan sulfate Gal-6 sulfotransferase
2	Efcab6L-like(HPU_09063)	Non_apical_ecto, Veg1_2_endo	EF-hand calcium binding domain 6-like
2	HscbL(HPU_00965)	Non_apical_ecto, Veg1_2_endo	similar to co-chaperone protein HscB, mitochondrial precursor
2	Ankdd1a(HPU_03065)	Non_apical_ecto, Veg1_ecto	ankyrin repeat and death domain containing 1A; ankyrin-like
2	C1orf177L(HPU_17172)	Non_apical_ecto, Veg1_ecto	human chromosome 1 open reading frame 177-like
2	Ckap2L(HPU_08364)	Non_apical_ecto, Veg1_ecto	cytoskeleton associated protein 2-like
2	Hypp_0466(HPU_01673)	Non_apical_ecto, Veg1_ecto	hypothetical protein-309
2	Hypp_2492(HPU_08871)	Non_apical_ecto, Veg1_ecto	none
2	Hypp_4451(HPU_15560)	Non_apical_ecto, Veg1_ecto	hypothetical protein-838
2	Hypp_5292(HPU_18270)	Non_apical_ecto, Veg1_ecto	none
2	Nacha3(HPU_06958)	Non_apical_ecto, Veg1_ecto	nicotinic acetylcholine receptor alpha 3
2	Nin(HPU_02484)	Non_apical_ecto, Veg1_ecto	ninein
2	Slc16a6L_11(HPU_24454)	Non_apical_ecto, Veg1_ecto	solute carrier family 16 (monocarboxylic acid transporters), member 6-like-11
2	Slc7a4_3(HPU_09810)	Non_apical_ecto, Veg1_ecto	Cationic amino acid transporter-4 (CAT-4) (CAT4) aka (14250696) Solute carrier family 7 (cationic amino acid transporter, y+ system), member 4
2	SpnprA(HPU_15870)	Non_apical_ecto, Veg1_ecto	none
2	Trpm_1(HPU_11724)	Non_apical_ecto, Veg1_ecto	transient receptor potential cation channel
2	Z421-like(HPU_15213)	Non_apical_ecto, Veg1_ecto	none
2	Cav1(HPU_03954)	Skeletogenic, Veg1_2_endo	none
2	Hypp_3079(HPU_10845)	Skeletogenic, Veg1_2_endo	none
2	Hypp_7350(HPU_25025)	Skeletogenic, Veg1_2_endo	none
2	Timp1b(HPU_11236)	Skeletogenic, Veg1_2_endo	tissue inhibitor of metalloproteases
2	Ust_2(HPU_12078)	Skeletogenic, Veg1_2_endo	uronyl-2-sulfotransferase
2	Rspo3(HPU_03672)	Veg1_2_endo, Veg1_ecto	R-spondin 3; thrombospondin
3	Ksr2(HPU_03576)	Apical_ecto, Germ_line, NSM	kinase suppressor of Ras 2
3	AnubL1(HPU_07000)	Apical_ecto, NSM, Veg1_ecto	AN1, ubiquitin-like, homolog (Xenopus laevis); ubiquitin-like fusion protein
3	Cabin1L(HPU_25000)	Apical_ecto, Non_apical_ecto, Veg1_2_endo	calcineurin binding protein 1-like
3	Ism(HPU_15654)	Apical_ecto, Non_apical_ecto, Veg1_2_endo	none
3	Adprh_2(HPU_12593)	Apical_ecto, Non_apical_ecto, Veg1_ecto	ADP-ribosylarginine hydrolase-2
3	Akap14(HPU_01826)	Apical_ecto, Non_apical_ecto, Veg1_ecto	A kinase (PRKA) anchor protein 14
3	Atub2(HPU_24328)	Apical_ecto, Non_apical_ecto, Veg1_ecto	none
3	C20orf103L(HPU_04400)	Apical_ecto, Non_apical_ecto, Veg1_ecto	human chromosome 20 open reading frame 103-like; lysosomal membrane glycoprotein A-like
3	Chst11L_1(HPU_11344)	Apical_ecto, Non_apical_ecto, Veg1_ecto	carbohydrate (chondroitin 4) sulfotransferase 11-like-1
3	Hypp_0164(HPU_00590)	Apical_ecto, Non_apical_ecto, Veg1_ecto	none
3	Hypp_0958(HPU_03572)	Apical_ecto, Non_apical_ecto, Veg1_ecto	hypothetical protein-2673
3	Hypp_3140(HPU_11058)	Apical_ecto, Non_apical_ecto, Veg1_ecto	none
3	Hypp_4426(HPU_15469)	Apical_ecto, Non_apical_ecto, Veg1_ecto	hypothetical protein-354
3	Hypp_6995(HPU_23910)	Apical_ecto, Non_apical_ecto, Veg1_ecto	none
3	Hypp_7268(HPU_24717)	Apical_ecto, Non_apical_ecto, Veg1_ecto	hypothetical protein-2603
3	Kiaa1529L(HPU_05474)	Apical_ecto, Non_apical_ecto, Veg1_ecto	KIAA1529-like
3	Rgs22L_1(HPU_23806)	Apical_ecto, Non_apical_ecto, Veg1_ecto	regulator of G-protein signaling 22-like-1
3	Rhogap20-like(HPU_10300)	Apical_ecto, Non_apical_ecto, Veg1_ecto	Rho GTPase activating protein 20, ARHGAP20, RA RhoGAP
3	Srcr97(HPU_03360)	Apical_ecto, Non_apical_ecto, Veg1_ecto	none
3	Tsnaxip1(HPU_15193)	Apical_ecto, Non_apical_ecto, Veg1_ecto	translin-associated factor X (Tsnax)-interacting protein 1
3	Z398-like(HPU_04039)	Apical_ecto, Non_apical_ecto, Veg1_ecto	none
3	Rdh12_2(HPU_12090)	Germ_line, NSM, Non_apical_ecto	retinol dehydrogenase 12_2
3	Asns_4(HPU_05878)	Germ_line, NSM, Skeletogenic	Asparaginyl-tRNA synthetase
3	GadD45(HPU_13495)	Germ_line, NSM, Skeletogenic	Growth Arrest and DNA-damage 45
3	Hypp_5706(HPU_19716)	Germ_line, NSM, Skeletogenic	none
3	Mae(HPU_19175)	Germ_line, NSM, Skeletogenic	none
3	Npnt(HPU_02856)	Germ_line, NSM, Skeletogenic	nephronectin
3	Plekha1(HPU_16193)	Germ_line, NSM, Skeletogenic	pleckstrin homology domain containing family A member 1
3	SmtnL(HPU_19908)	Germ_line, NSM, Skeletogenic	smoothelin-like
3	Dhx37(HPU_17310)	Germ_line, NSM, Veg1_2_endo	DEAH (Asp-Glu-Ala-His) box polypeptide 37
3	Hypp_0973(HPU_03634)	Germ_line, NSM, Veg1_2_endo	none
3	PppL_224(HPU_03117)	Germ_line, NSM, Veg1_2_endo	pol polyprotein like-224
3	Ddx31(HPU_16689)	Germ_line, Non_apical_ecto, Veg1_2_endo	DEAD (Asp-Glu-Ala-Asp) box polypeptide 31
3	Dhx29(HPU_04058)	Germ_line, Non_apical_ecto, Veg1_2_endo	DEAH (Asp-Glu-Ala-His) box polypeptide 29
3	EchnL_13-like(HPU_06717)	Germ_line, Non_apical_ecto, Veg1_2_endo	echinonectin-like-13
3	Hypp_2973(HPU_10495)	Germ_line, Non_apical_ecto, Veg1_2_endo	none
3	Uckl1(HPU_09556)	Germ_line, Non_apical_ecto, Veg1_2_endo	Uridine/cytidine kinase-like 1
3	Wwc1(HPU_08365)	Germ_line, Non_apical_ecto, Veg1_2_endo	WW, C2 and coiled-coil domain containing 1
3	Hypp_6206(HPU_21348)	Germ_line, Non_apical_ecto, Veg1_ecto	hypothetical protein-1146; myeloid leukemia factor 1 interacting protein-like
3	Lhh2ag(HPU_18200)	Germ_line, Non_apical_ecto, Veg1_ecto	none
3	Tbrg4(HPU_10736)	Germ_line, Skeletogenic, Veg1_2_endo	transforming growth factor beta regulator 4
3	Krfp1L(HPU_17947)	Germ_line, Skeletogenic, Veg1_ecto	krev interaction trapped 1 isoform 2, KRIT1 ankyrin repeat containing
3	Abcd3(HPU_15253)	NSM, Non_apical_ecto, Skeletogenic	none
3	Polyd(HPU_05294)	NSM, Non_apical_ecto, Skeletogenic	sushi, von Willebrand factor type A, EGF and pentraxin domain containing 1, human SEL-OB
3	Hypp_4657(HPU_16198)	NSM, Non_apical_ecto, Veg1_2_endo	none
3	Hypp_0215(HPU_00752)	NSM, Non_apical_ecto, Veg1_ecto	none
3	Galnt13(HPU_17710)	NSM, Skeletogenic, Veg1_2_endo	UDP-N-acetyl-alpha-D-galactosamine:polypeptide N-acetylgalactosaminyltransferase 13
3	Hypp_1598(HPU_05798)	NSM, Skeletogenic, Veg1_2_endo	none
3	Chmp6(HPU_07676)	NSM, Skeletogenic, Veg1_ecto	Charged multivesicular body protein 6, Chromatin-modifying protein 6
3	Hypp_6378(HPU_21940)	NSM, Skeletogenic, Veg1_ecto	hypothetical protein-1597; serine/threonine protein phosphatase-like
3	Camsap1L(HPU_04857)	Non_apical_ecto, Skeletogenic, Veg1_ecto	calmodulin regulated spectrin-associated protein 1-like
3	Cyp27L(HPU_10279)	Non_apical_ecto, Skeletogenic, Veg1_ecto	Cytochrome P450 Family 27 like, CYP27
3	Il16L(HPU_21595)	Non_apical_ecto, Skeletogenic, Veg1_ecto	interleukin 16 like
3	C3-2(HPU_03501)	Non_apical_ecto, Veg1_2_endo, Veg1_ecto	none
3	Ctr1aL(HPU_17512)	Non_apical_ecto, Veg1_2_endo, Veg1_ecto	copper transporter 1A (Drosophila)-like
3	Cyp1r4(HPU_10316)	Non_apical_ecto, Veg1_2_endo, Veg1_ecto	cytochrome P450 Family 1 related partial, CYP2 , CYP1-related
3	Hypp_4124(HPU_14432)	Non_apical_ecto, Veg1_2_endo, Veg1_ecto	hypothetical protein-1374; transmembrane protein 132C-like
3	Hypp_6087(HPU_20968)	Non_apical_ecto, Veg1_2_endo, Veg1_ecto	none
3	Nkain1(HPU_08162)	Non_apical_ecto, Veg1_2_endo, Veg1_ecto	Na+/K+ transporting ATPase interacting 1; NKAIN1 protein
3	P75ntr(HPU_15169)	Non_apical_ecto, Veg1_2_endo, Veg1_ecto	p75 Neurotrophin Receptor
3	Slc5a12(HPU_12788)	Non_apical_ecto, Veg1_2_endo, Veg1_ecto	solute carrier family 5 (sodium/glucose cotransporter), member 12 (33328174) Na-I related cotransporter
3	Wif(HPU_07446)	Non_apical_ecto, Veg1_2_endo, Veg1_ecto	Wnt inhibitory factor 1
3	blaZ(HPU_02689)	Non_apical_ecto, Veg1_2_endo, Veg1_ecto	beta-lactamase (bacterial)
4	Kiaa1598L(HPU_20367)	Apical_ecto, Germ_line, NSM, Veg1_ecto	KIAA1598 protein-like
4	Fam120c(HPU_03280)	Apical_ecto, Germ_line, Non_apical_ecto, Veg1_2_endo	family with sequence similarity 120C
4	Hypp_0374(HPU_01313)	Apical_ecto, Germ_line, Non_apical_ecto, Veg1_2_endo	none
4	Hypp_1762(HPU_06352)	Apical_ecto, Germ_line, Non_apical_ecto, Veg1_2_endo	hypothetical protein-14; BAH domain and coiled-coil containing 1-like; zinc finger protein 469-like
4	Ris(HPU_16407)	Apical_ecto, Germ_line, Non_apical_ecto, Veg1_2_endo	Ras-like family 12, RASL12
4	Tlk(HPU_16240)	Apical_ecto, Germ_line, Non_apical_ecto, Veg1_2_endo	Tousled-like kinase
4	Costal2(HPU_24820)	Apical_ecto, Germ_line, Non_apical_ecto, Veg1_ecto	KIF27, KIF7
4	Diversin(HPU_12518)	Apical_ecto, Germ_line, Non_apical_ecto, Veg1_ecto	ankyrin repeat domain 6
4	Hypp_1930(HPU_07002)	Apical_ecto, Germ_line, Non_apical_ecto, Veg1_ecto	hypothetical protein-552
4	Hypp_4249(HPU_14867)	Apical_ecto, Germ_line, Non_apical_ecto, Veg1_ecto	hypothetical protein-871
4	Hypp_6280(HPU_21615)	Apical_ecto, Germ_line, Non_apical_ecto, Veg1_ecto	none
4	Hypp_7158(HPU_24381)	Apical_ecto, Germ_line, Non_apical_ecto, Veg1_ecto	hypothetical protein-2392; coiled-coil domain containing 74B-like
4	Wd66(HPU_17331)	Apical_ecto, Germ_line, Non_apical_ecto, Veg1_ecto	WD repeat domain 66
4	Lrp6l(HPU_10967)	Apical_ecto, NSM, Non_apical_ecto, Veg1_ecto	LDL receptor-related protein 6
4	Sarmr4(HPU_01784)	Apical_ecto, Non_apical_ecto, Skeletogenic, Veg1_ecto	Sp-Sarm-related 4
4	Accn1_1(HPU_22480)	Apical_ecto, Non_apical_ecto, Veg1_2_endo, Veg1_ecto	Amiloride-sensitive cation channel 1, neuronal (Amiloride-sensitive brain sodium channel) (Brain sodium channel 1) (BNaC1) (Amiloride-sensitive cation channel neuronal 1) (BNC1) (Degenerin channel MDEG
4	Efcab6L_2-like(HPU_02513)	Apical_ecto, Non_apical_ecto, Veg1_2_endo, Veg1_ecto	EF-hand calcium binding domain 6-like-2
4	Gal3st1-3(HPU_24866)	Apical_ecto, Non_apical_ecto, Veg1_2_endo, Veg1_ecto	galactose-3-O-sulfotransferase 1-3
4	Hypp_3128(HPU_11009)	Apical_ecto, Non_apical_ecto, Veg1_2_endo, Veg1_ecto	none
4	Hypp_6997(HPU_23916)	Apical_ecto, Non_apical_ecto, Veg1_2_endo, Veg1_ecto	none
4	Acly_2(HPU_00328)	Germ_line, NSM, Non_apical_ecto, Skeletogenic	ATP-citrate synthase (ATP-citrate (pro-S-)-lyase) (Citrate cleavage enzyme)
4	Agpat1_2(HPU_22331)	Germ_line, NSM, Non_apical_ecto, Skeletogenic	1-acyl-sn-glycerol-3-phosphate acyltransferase alpha (1-AGP acyltransferase 1) (1-AGPAT 1) (Lysophosphatidic acid acyltransferase-alpha) (LPAAT-alpha) (1-acylglycerol-3-phosphate O-acyltransferase 1) (G15 protein)
4	Hypp_1015(HPU_03772)	Germ_line, NSM, Non_apical_ecto, Skeletogenic	hypothetical protein-258
4	Hypp_0613(HPU_02273)	Germ_line, NSM, Non_apical_ecto, Veg1_2_endo	hypothetical protein-2877
4	Hypp_5920(HPU_20369)	Germ_line, NSM, Non_apical_ecto, Veg1_2_endo	hypothetical protein-625
4	Runx1t1(HPU_18779)	Germ_line, NSM, Non_apical_ecto, Veg1_2_endo	runt-related transcription factor 1; translocated to, 1 (cyclin D-related); core-binding factor, runt domain, alpha subunit 2 - translocated to, 3; acute myelogenous leukemia 1 translocation 1 protein
4	Unk_16(HPU_14923)	Germ_line, NSM, Non_apical_ecto, Veg1_2_endo	U2 small nuclear RNA auxillary factor 1 isoform a
4	Hypp_0038(HPU_00162)	Germ_line, NSM, Non_apical_ecto, Veg1_ecto	hypothetical protein-1654
4	Lhh2bg(HPU_18714)	Germ_line, NSM, Non_apical_ecto, Veg1_ecto	none
4	AcpL2(HPU_04643)	Germ_line, NSM, Skeletogenic, Veg1_2_endo	acid phosphatase-like protein
4	Chst1(HPU_06770)	Germ_line, NSM, Skeletogenic, Veg1_2_endo	carbohydrate sulfotransferase 1, keratan sulfate Gal-6 sulfotransferase
4	Hypp_4688(HPU_16280)	Germ_line, NSM, Skeletogenic, Veg1_2_endo	none
4	Pdgfr/vegfrL(HPU_15527)	Germ_line, NSM, Skeletogenic, Veg1_2_endo	none
4	Hypp_1757(HPU_06338)	Germ_line, NSM, Skeletogenic, Veg1_ecto	none
4	Trrspn_19(HPU_14013)	Germ_line, NSM, Skeletogenic, Veg1_ecto	none
4	Tkt(HPU_15621)	Germ_line, NSM, Veg1_2_endo, Veg1_ecto	Transketolase (TK)
4	Dnl3(HPU_17529)	Germ_line, Non_apical_ecto, Skeletogenic, Veg1_2_endo	DNA ligase III (Polydeoxyribonucleotide synthase [ATP])
4	Slc45a2_1(HPU_03376)	Germ_line, Non_apical_ecto, Skeletogenic, Veg1_ecto	Membrane-associated transporter protein
4	Hypp_6440(HPU_22157)	Germ_line, Non_apical_ecto, Veg1_2_endo, Veg1_ecto	hypothetical protein-537
4	Kiaa1731L(HPU_07577)	Germ_line, Non_apical_ecto, Veg1_2_endo, Veg1_ecto	eucine-rich repeat-containing protein KIAA1731-like
4	Aak1-like(HPU_10014)	NSM, Non_apical_ecto, Skeletogenic, Veg1_2_endo	AP2 associated kinase
4	Mphosph6(HPU_21409)	NSM, Non_apical_ecto, Veg1_2_endo, Veg1_ecto	M phase phosphoprotein 6
4	Cyp2L52(HPU_04729)	Non_apical_ecto, Skeletogenic, Veg1_2_endo, Veg1_ecto	Cytochrome P450 Family 2 like, CYP2
5	Adam/TsL6(HPU_07529)	Apical_ecto, Germ_line, NSM, Non_apical_ecto, Veg1_2_endo	none
5	Grwd1(HPU_00048)	Apical_ecto, Germ_line, NSM, Non_apical_ecto, Veg1_2_endo	glutamate-rich WD repeat containing 1
5	Hypp_4786(HPU_16604)	Apical_ecto, Germ_line, NSM, Non_apical_ecto, Veg1_2_endo	none
5	Netrin1(HPU_01990)	Apical_ecto, Germ_line, NSM, Non_apical_ecto, Veg1_2_endo	none
5	Wdr74(HPU_11045)	Apical_ecto, Germ_line, NSM, Non_apical_ecto, Veg1_2_endo	WD repeat domain 74; NOP seven associated protein 1
5	Kiaa1751L(HPU_04670)	Apical_ecto, Germ_line, Non_apical_ecto, Skeletogenic, Veg1_ecto	KIAA1751 protein-like
5	Cdc20(HPU_10251)	Apical_ecto, Germ_line, Non_apical_ecto, Veg1_2_endo, Veg1_ecto	Cell division cycle protein 20 homolog
5	Gal/Lect/Lnb/7Tm/Gpcr_1(HPU_13535)	Apical_ecto, Germ_line, Non_apical_ecto, Veg1_2_endo, Veg1_ecto	none
5	GtpbpL2(nucleolar)-like(HPU_08550)	Apical_ecto, Germ_line, Non_apical_ecto, Veg1_2_endo, Veg1_ecto	none
5	Hsp40A(HPU_12003)	Apical_ecto, Germ_line, Non_apical_ecto, Veg1_2_endo, Veg1_ecto	DNAJ
5	Hypp_2681(HPU_09495)	Apical_ecto, Germ_line, Non_apical_ecto, Veg1_2_endo, Veg1_ecto	none
5	Hypp_3516(HPU_12425)	Apical_ecto, Germ_line, Non_apical_ecto, Veg1_2_endo, Veg1_ecto	hypothetical protein-992
5	Hypp_3845(HPU_13497)	Apical_ecto, Germ_line, Non_apical_ecto, Veg1_2_endo, Veg1_ecto	none
5	Kndc1(HPU_16094)	Apical_ecto, Germ_line, Non_apical_ecto, Veg1_2_endo, Veg1_ecto	kinase non-catalytic C-lobe domain (KIND) containing 1
5	Lhh2ac(HPU_18886)	Apical_ecto, Germ_line, Non_apical_ecto, Veg1_2_endo, Veg1_ecto	none
5	Lrrc23(HPU_16488)	Apical_ecto, Germ_line, Non_apical_ecto, Veg1_2_endo, Veg1_ecto	leucine rich repeat containing 23
5	Pkd2(HPU_05763)	Apical_ecto, Germ_line, Non_apical_ecto, Veg1_2_endo, Veg1_ecto	suPKD2, polycystin-2, suPC2
5	Ppie_1(HPU_16330)	Apical_ecto, Germ_line, Non_apical_ecto, Veg1_2_endo, Veg1_ecto	peptidylprolyl isomerase E
5	Hrh1_1(HPU_01186)	Apical_ecto, NSM, Non_apical_ecto, Veg1_2_endo, Veg1_ecto	histamine receptor H 1-1
5	Hypp_7075(HPU_24120)	Apical_ecto, NSM, Non_apical_ecto, Veg1_2_endo, Veg1_ecto	none
5	Phtf2L(HPU_24645)	Germ_line, NSM, Non_apical_ecto, Skeletogenic, Veg1_2_endo	putative homeodomain transcription factor 2-like
5	Hypp_1961(HPU_07108)	Germ_line, NSM, Non_apical_ecto, Skeletogenic, Veg1_ecto	hypothetical protein-2120; superoxide dismutase-like
5	Olah(HPU_19490)	Germ_line, NSM, Non_apical_ecto, Skeletogenic, Veg1_ecto	oleoyl-ACP hydrolase; thioesterase domain containing 1
5	Lhh2aa(HPU_16053)	Germ_line, NSM, Non_apical_ecto, Veg1_2_endo, Veg1_ecto	none
5	Glt_3(HPU_04281)	NSM, Non_apical_ecto, Skeletogenic, Veg1_2_endo, Veg1_ecto	glycosyl transferase family protein (bacterial)
6	FormL2iB-like(HPU_02903)	Apical_ecto, Germ_line, NSM, Non_apical_ecto, Skeletogenic, Veg1_2_endo	none
6	Hypp_4046(HPU_14132)	Apical_ecto, Germ_line, NSM, Non_apical_ecto, Skeletogenic, Veg1_ecto	hypothetical protein-1687
6	Atpif1L(HPU_24643)	Apical_ecto, Germ_line, NSM, Non_apical_ecto, Veg1_2_endo, Veg1_ecto	ATPase inhibitory factor 1 like
6	Ddx18(HPU_18080)	Apical_ecto, Germ_line, NSM, Non_apical_ecto, Veg1_2_endo, Veg1_ecto	DEAD (Asp-Glu-Ala-Asp) box polypeptide 18
6	Hypp_4103(HPU_14351)	Apical_ecto, Germ_line, NSM, Non_apical_ecto, Veg1_2_endo, Veg1_ecto	none
6	Hypp_5919(HPU_20368)	Apical_ecto, Germ_line, NSM, Non_apical_ecto, Veg1_2_endo, Veg1_ecto	none
6	Mdh1(HPU_09790)	Apical_ecto, Germ_line, NSM, Non_apical_ecto, Veg1_2_endo, Veg1_ecto	Malate dehydrogenase, cytoplasmic
6	Rbm17L(HPU_23443)	Apical_ecto, Germ_line, NSM, Non_apical_ecto, Veg1_2_endo, Veg1_ecto	RNA binding motif protein 17
6	U2A-like(HPU_02551)	Apical_ecto, Germ_line, NSM, Non_apical_ecto, Veg1_2_endo, Veg1_ecto	U2 small nuclear ribonucleoprotein A (U2 snRNP-A)
6	A2ml1L-like(HPU_16903)	Apical_ecto, Germ_line, Non_apical_ecto, Skeletogenic, Veg1_2_endo, Veg1_ecto	alpha-2-macroglobulin-like 1-like
6	Hypp_3031(HPU_10724)	Apical_ecto, Germ_line, Non_apical_ecto, Skeletogenic, Veg1_2_endo, Veg1_ecto	none
6	Hypp_7069(HPU_24110)	Apical_ecto, Germ_line, Non_apical_ecto, Skeletogenic, Veg1_2_endo, Veg1_ecto	hypothetical protein-1543
6	Odin/CajL-like(HPU_05729)	Apical_ecto, Germ_line, Non_apical_ecto, Skeletogenic, Veg1_2_endo, Veg1_ecto	none
6	Spag1(HPU_24181)	Apical_ecto, Germ_line, Non_apical_ecto, Skeletogenic, Veg1_2_endo, Veg1_ecto	sperm associated antigen 1
6	Tmem49(HPU_13417)	Apical_ecto, Germ_line, Non_apical_ecto, Skeletogenic, Veg1_2_endo, Veg1_ecto	transmembrane protein 49
6	Hypp_0344(HPU_01210)	Apical_ecto, NSM, Non_apical_ecto, Skeletogenic, Veg1_2_endo, Veg1_ecto	none
6	Hypp_1679(HPU_06091)	Apical_ecto, NSM, Non_apical_ecto, Skeletogenic, Veg1_2_endo, Veg1_ecto	none
6	Sh3gl3(HPU_16409)	Apical_ecto, NSM, Non_apical_ecto, Skeletogenic, Veg1_2_endo, Veg1_ecto	Endophilin III
6	Adam/Ts6/10L1(HPU_23964)	Germ_line, NSM, Non_apical_ecto, Skeletogenic, Veg1_2_endo, Veg1_ecto	none
6	Creb3l1(HPU_14522)	Germ_line, NSM, Non_apical_ecto, Skeletogenic, Veg1_2_endo, Veg1_ecto	none
6	Cycpln(HPU_14178)	Germ_line, NSM, Non_apical_ecto, Skeletogenic, Veg1_2_endo, Veg1_ecto	none
6	Golga1L(HPU_19916)	Germ_line, NSM, Non_apical_ecto, Skeletogenic, Veg1_2_endo, Veg1_ecto	golgi autoantigen, golgin subfamily a, 1-like
6	Irsp53/58(HPU_16737)	Germ_line, NSM, Non_apical_ecto, Skeletogenic, Veg1_2_endo, Veg1_ecto	Insulin receptor substrate p53/p58 - IRSp53/58 - Fas ligand-associated factor 3 - FLAF3 - BAIAP2 homolog
6	Msp130(HPU_05528)	Germ_line, NSM, Non_apical_ecto, Skeletogenic, Veg1_2_endo, Veg1_ecto	none
6	Muc4-like(HPU_01013)	Germ_line, NSM, Non_apical_ecto, Skeletogenic, Veg1_2_endo, Veg1_ecto	mucin 4, cell surface associated
6	Parp1(HPU_04651)	Germ_line, NSM, Non_apical_ecto, Skeletogenic, Veg1_2_endo, Veg1_ecto	Poly [ADP-ribose] polymerase 1
6	Parp3(HPU_02616)	Germ_line, NSM, Non_apical_ecto, Skeletogenic, Veg1_2_endo, Veg1_ecto	Poly [ADP-ribose] polymerase 3
6	Pdia5(HPU_06249)	Germ_line, NSM, Non_apical_ecto, Skeletogenic, Veg1_2_endo, Veg1_ecto	Protein disulfide-isomerase A5 precursor (Protein disulfide isomerase-related protein)
6	Pofut1-like(HPU_08250)	Germ_line, NSM, Non_apical_ecto, Skeletogenic, Veg1_2_endo, Veg1_ecto	protein-O-fucosyltransferase 1, OFUT1, OFUCT1
6	Scube(HPU_20921)	Germ_line, NSM, Non_apical_ecto, Skeletogenic, Veg1_2_endo, Veg1_ecto	signal peptide,CUB domain,EGF-like/CEGP
6	Slc24a6-like(HPU_23091)	Germ_line, NSM, Non_apical_ecto, Skeletogenic, Veg1_2_endo, Veg1_ecto	solute carrier family 24 member 6
6	Slco4a1_3(HPU_08740)	Germ_line, NSM, Non_apical_ecto, Skeletogenic, Veg1_2_endo, Veg1_ecto	Solute carrier organic anion transporter family member 4A1 (Solute carrier family 21 member 12) (Sodium-independent organic anion transporter E) (Organic anion transporting polypeptide E) (OATP-E) (Colon organic aniontransporter) (Organic anion transporter polypeptide-related protein 1) (OATP-RP1) (OATPRP1) (POAT)
6	Sm30A(HPU_12534)	Germ_line, NSM, Non_apical_ecto, Skeletogenic, Veg1_2_endo, Veg1_ecto	none
6	Thada(HPU_18756)	Germ_line, NSM, Non_apical_ecto, Skeletogenic, Veg1_2_endo, Veg1_ecto	thyroid adenoma associated; death receptor interacting/3p fusion protein
6	UacaL(HPU_21769)	Germ_line, NSM, Non_apical_ecto, Skeletogenic, Veg1_2_endo, Veg1_ecto	uveal autoantigen with coiled-coil domains and ankyrin-like
6	Usp30(HPU_08859)	Germ_line, NSM, Non_apical_ecto, Skeletogenic, Veg1_2_endo, Veg1_ecto	ubiquitin specific protease 30
6	p58-a(HPU_22824)	Germ_line, NSM, Non_apical_ecto, Skeletogenic, Veg1_2_endo, Veg1_ecto	none
